# Remodeling of tumor microenvironment for enhanced tumor chemodynamic/photothermal/chemo-therapy

**DOI:** 10.1186/s12951-022-01594-4

**Published:** 2022-08-26

**Authors:** Ying Zhang, Jingyao Zhu, Zheng Zhang, Dannong He, Jun Zhu, Yunsheng Chen, Yixin Zhang

**Affiliations:** 1 Department of Plastic and Reconstructive Surgery, Shanghai Ninth People’s Hospital, Shanghai Jiao Tong University School of Medicine, 639 Zhizaoju Road, Shanghai, 200011 People’s Republic of China; 2 School of Materials Science and Engineering, Shanghai Jiao Tong University, Shanghai, 200240 People’s Republic of China; 3 National Engineering Research Center for Nanotechnology, Shanghai, 200241 People’s Republic of China; 4 Shanghai Burns Institute, Rui Jin Hospital, Shanghai Jiao Tong University School of Medicine, No.197, Rui Jin 2nd Road, Shanghai, 200025 China

**Keywords:** Vascular normalization, Tumor microenvironment, Chemodynamic therapy, Thermo/chemo-therapy, Hyperoxygen

## Abstract

The anticancer treatment is largely affected by the microenvironment of the tumors, which not only resists the tumors to the thermo/chemo-therapy, but also promotes their growth and invasion. In this work, the angiogenesis factor is balanced by combining with the breathing hyperoxygen, for regulating the tumor microenvironment and also for relieving hypoxia and high tissue interstitial pressure, which promote drug delivery to tumor tissues by increasing the in vivo perfusion and reversing the immunosuppressive tumor. In addition, the designed multifunctional nanoparticles have a great potential for applications to the tumor dual-mode imaging including magnetic resonance (MR) and photoacoustic (PA) imaging. This work proposes a promising strategy to enhance the thermo/chemo-therapy efficacy by remodeling the tumor microenvironment, which would provide an alternative to prolong the lifetime of tumor patients.

**Graphical Abstract:**

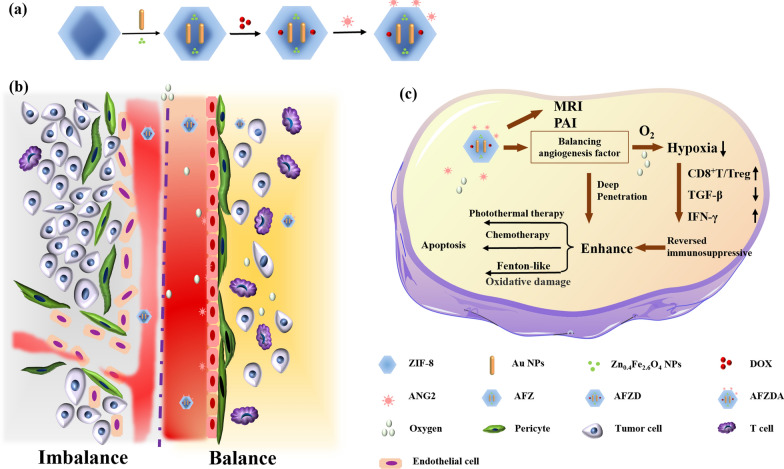

## Introduction

The thermo/chemo-therapy is proven to be effective in cancer treatments. However, the efficient delivery and penetration of therapeutic agents into a solid tumor remain a thorny problem [1, 2]. Previous studies showed that the blocking of angiogenesis can slow down the tumor growth, but it may paradoxically increase the tumor metastasis [3]. In tumor tissues where the vascular regulation imbalance exists [4, 5], the related cytokines Angiogenin-2 (ANG-2) and vascular endothelial growth factor (VEGF) are overexpressed to promote the pathological angiogenesis particularly in the early period of the tumor progression, which are immature and leaky blood vessels [6, 7]. It is found that abnormal tumor blood vessels lead to high interstitial pressure [8, 9] and poor blood perfusion in tumor tissues [10]. The deficiency in the vessel structures and functions is a major factor that impedes the delivery of thermo/chemo-therapeutic materials [11, 12]. However, the traditional antiangiogenesis often leads to extreme hypoxia in tumors through vascular regression, and prevents the anti-tumor immune cells from further entering into the tumor tissues [13–15]. Moreover, the hypoxia enhances invasion and metastasis by making the endothelial cells of the distal organ vulnerable to the angiogenesis factors [16–19]. The hypoxia also blocks the autoimmunization, which carries a risk of the continued deterioration of the tumors [20–22]. The relief of the hypoxia can overcome the multidrug resistance and immunosuppression to thermo/chemo-therapy [23–25].

The vascular normalization is attracting increasing attention to tumor therapy as it can change the intratumoral microenvironment and reduce the hypoxia, which overcomes multidrug resistance and immunosuppression and favors thermo/chemo-therapy [26, 27]. The Anti-ANG-2 and Anti-VEGF antibodies can repair tumor vascular loopholes, increase vascular stability, and suppress tumor angiogenesis [28, 29]. On the other hand, reduced interstitial fluid pressure (IFP) and increased the perfusion ability of blood in the tumors are beneficial for drugs and immune effector cells through blood delivery as far as the distal end [17, 30, 31]. In light of these, a promising strategy is the provision of favorable conditions for thermochemo-therapy [32]. The nanotechnology can be used to improve the tumor microenvironment by alleviating immune inhibition and increasing oxygen concentration in the tumors [33].

Those nanoparticles, which are capable of regulating the tumor microenvironment and collaborative treatment play a key role in improving drug utilization and reducing side effects. In addition, clinical imaging techniques such as the photoacoustic imaging (PAI), magnetic resonance imaging (MRI), computerized tomography (CT), and optical imaging, are used extensively to monitor the changes in the tumor microenvironment (TME) resulting from an anti-tumor therapy [34, 35]. Magnetic ferri-based oxides have good magnetic resonance imaging capability, and Au nanorods can realize near-infrared photothermal conversion and photoacoustic imaging, which can be utilized in visual monitoring [36, 37].

In the present work, a core–shell structured drug-loading nanoframe of the form of functional modification nanoparticles is prepared that can enhance the chemodynamic therapy and thermo/chemo-therapy efficacy for tumors based on vascular normalization and reverse immunosuppressive TME. Therefore, drug delivery and anti-tumor immune cell infiltration to tumor tissues would be promoted [38]. Moreover, the multifunctional nanoparticles have great potential for applications to the tumor dual-mode imaging, such as MRI and PAI. This work proposes an effective strategy to improve the tumor microenvironment and normalize abnormal vascular properties. The developed multifunctional nanoparticles can optimize the thermo/chemo-therapy and achieve real-time dual-mode imaging.

## Materials and methods

### Materials

Chloroauric acid was supplied by Sigma-Aldrich Co., Ltd. Phosphate Buffered Saline (PBS) pH = 7.4, silver nitrate (AgNO_3_), zinc sulfate (ZnSO_4_), sodium borohydride (NaBH_4_), oleic acid, sodium hydroxide (NaOH), chloroform, FeSO_4_·(NH_4_)_2_SO_4_·6H_2_O, and triethylamine was supplied by Sinopharm chemical reagent Co., Ltd. Oleic acid, ethanol and formaldehyde (37–40 wt%) were purchased from Shanghai Titan Scientific Co., Ltd. Fluorescein isothiocyanate and doxorubicin hydrochloride was purchased from Aladdin Co., Ltd. Dimethyl sulfoxide(DMSO) and dimercaptosuccinic acid (DMSA) was supplied by Adamas Co., Ltd. VEGF165 angiogenesis cytokine, Anti-angiogenin 2, Anti-VEGF was purchased Abcam. Deionized water was used during the experiment from Millipore water purification system (Merck, Plus185). All reagents were not further purified.

### Zn_0.4_Fe_2.6_O_4_ NPs synthesis

Firstly, Zn_0.4_Fe_2.6_O_4_ NPs capped by oleic acid were prepared. Specifically, 1.37 g of FeSO_4_· (NH_4_)_2_SO_4_·6H_2_O and 0.54 g of ZnSO_4_ were dissolved in 40 mL of water. 2 g of sodium hydroxide, 20 mL of oleic acid, and 20 mL of ethanol were mixed and stirred to obtain a homogeneous solution. Subsequently, the solution was transferred into a 100 mL reactor, and kept at 230 °C for 16 h. The product was washed with ethanol for three times, dispersed in chloroform, and stored at 4 °C. Then 100 mg of the product was dissolved in 10 mL of chloroform, and 50 μL of triethylamine was added. Thereafter, 50 mL of DMSO containing 50 mg of DMSA was added, and heated to 60 ℃, and stirred for 12 h. The precipitation was collected, and this procedure was repeated, followed by washing with ethanol three times to obtain water-soluble DMSA-modified Zn_0.4_Fe_2.6_O_4_ NPs.

### Preparation of Au/Zn_0.4_Fe_2.6_O_4_@Zif-8/DOX/anti-ANG2 (AFZDA) NPs

**Fig. 1:**
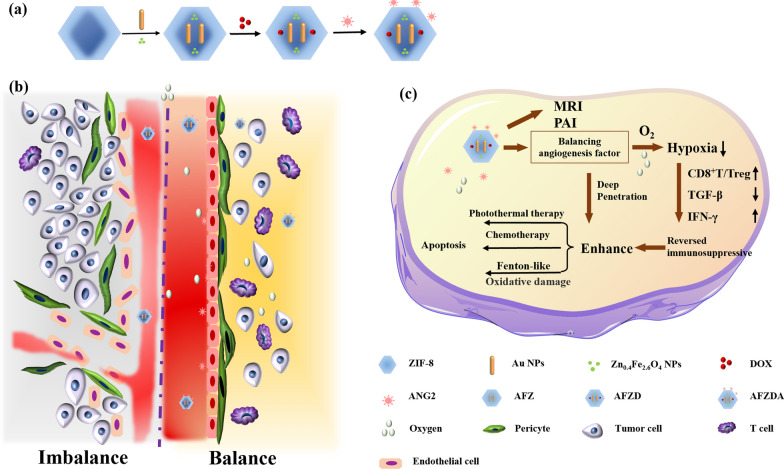
**a** The functionalization schemes of AFZDA NPs. **b** The schematic illustration of tumor vascular normalization. **C **AFZDA NPs for synergistic treatment

Au nanorods were prepared with a seedless growth method [39]. Au/Zn_0.4_Fe_2.6_O_4_@Zif-8 nanoparticles (AFZ NPs) were synthesized by a one-step solvothermal method. Typically, 50 mg of Au nanorods were dispersed in 100 mg of polyvinylpyrrolidone. Meanwhile, 0.42 g of zinc nitrate hexahydrate was dissolved in 20 mL of methanol/water (1:1, v/v) mixture. Then 1.0 g of 2-methylimidazole dissolved in 10 mL of methanol was added, and mixed at room temperature, kept for 4 h. The product Au/ Zn_0.4_Fe_2.6_O_4_@Zif-8 (AFZ) was centrifuged at 9000 rpm for 10 min, washed with anhydrous ethanol three times and dried in a vacuum. Thereafter, the obtained AFZ NPs (20 mg) were added to 10 mL of DOX (1.0 mg/mL) solution, stirred for 24 h, and the resultant nanoparticles were thoroughly washed by water, giving DOX-loading AFZD NPs. Finally, 10 mg of anti-ANG2 was mixed with 100 mL of AFZD NPs (1.0 mg/mL) aqueous solution under stirring for 2 h, and the anti-ANG2 was fixed on the surface of AFZD by electrostatic adsorption. The preparation strategy of AFZD NPs is shown in Fig. 1.

### Characterization

The structure of prepared nanoparticles was analyzed by field-emission transmission electron microscopy (TEM) from JEOL, model JEM-2010F, voltage 200 kV. The morphology and composition distribution were investigated by field-emission scanning electron microscopy (SEM) from Hitachi, equipped with an energy dispersive X-ray detector (EDX) from Oxford X-max 50. The size and zeta potential of prepared nanoparticles were measured by Malvern Zetasizer Nano ZS. The absorption spectra were measured via a PerkinElmer Ultraviolet spectrophotometer. The crystallographic structure of the nanoparticles was determined with the powder X-ray diffraction (XRD) from Rigaku, max-2600PC. The specific surface area and pore size distribution was collected via a Micromeritics ASAP 2020 instrument with Brunauer Emmett Teller (BET) method.

### Loading amount of DOX

To determine the loading amount of DOX, 20 mg of AFZ NPs was added to 10 mL of DOX (1.0 mg/mL) solution and stirred for 24 h. The unloaded DOX was separated from the resultant AFZD NPs by centrifugation and the amount was determined with an Ultraviolet spectrophotometer at about 490 nm (the absorption peak intensity is proportional to the concentration according to the standard curve of DOX aqueous solution). The loading amount of DOX was calculated by the following equation: loading amount = (W_total_−W_free_)/W_NP_, where W_total_ is the weight of fed DOX; W_free_ is the weight of free DOX; W_NP_ is the weight of the AFZD NPs.

### Loading amount of Anti-ANG2

To determine the loading amount of anti-ANG2, 10 mg of anti-ANG2 was added to 100 mL of AFZD (1.0 mg/mL) solution and stirred for 24 h. The unloaded anti-ANG2 was separated from the resultant AFZDA NPs by centrifugation and the concentration of anti-ANG2 in the supernatant was determined with an enzyme-linked immunosorbent assay. The amount of anti-ANG2 was calculated by the following equation: loading amount = (W_total_ − W_free_)W_NP_, where W_total_ is the amount of fed anti-ANG2; W_free_ is the weight of free anti-ANG2; W_NP_ is the weight of AFZDA NPs.

### Release of DOX

0.75 mg/mL AFZD NPs were lamped into permeable membranes in PBS of pH 6.4 and 7.0, respectively. The time-dependent DOX release from AFZD NPs was measured. The amount of released DOX was determined with an ultraviolet spectrophotometer at the wavelength of ~ 490 nm. The amount of DOX was calculated by the following equation: releasing amount = W_release_/W_load_ × 100%, where W_load_ is the weight of loaded DOX and W_free_ is the weight of released DOX;

### AFZD photothermal conversion efficiency

1 mL of AFZD nanoparticles aqueous solution in a quartz cuvette was irradiated by a near-infrared laser (808 nm, 1.5 W/cm^2^) for 10 min until reaching thermal equilibrium. And the laser source was shut down and the aqueous solution was allowed to cool down naturally. The rate of heat transfer was calculated from the system to the surrounding environment by regression of the cooling curve measured.

Following Roper‘s report, the photothermal conversion efficiency, η, was calculated using the following equation [40]:

1 $$\eta \, = \,\frac{{hS\left( {T_{max} - T_{surr} } \right) - Q_{dis} }}{{I\left( {1 - 10^{{ - A_{808} }} } \right)}}$$

In Equation, *T*_*max*_ is the maximum system temperature and *T*_*surr*_ is the surrounding temperature; *I* is incident laser power, and A808 is the OD value of AFZD solution at 808 nm. *Q*_*dis*_ is the baseline energy input from the light absorption by the solvent. *h* represents the heat transfer coefficient, *S* represents the surface area of the cuvette, and the value of *hS* is determined by the temperature reducing rate after turning off the light source, which can be calculated according to the following equations [41].

2 $${\text{hS}}\, = \,\frac{{mC_{p} }}{{{\uptau }_{{\text{s}}} }}$$

3 $${\text{t}}\, = \,{\uptau }_{{\text{s}}} {\text{ln}}\theta$$

4 $$\theta = \frac{{T - T_{surr} }}{{T_{max} - T_{surr} }}$$

m and C_p_ are the mass and the thermal capacity (aqueous solution of 4.2 J/g/°C was used); T is the temperature at the cooling time (t). The time constant τ_s_ was determined by linear fitting cooling time (t) to -lnθ.

### Cytotoxicity evaluation

The cytotoxicity was evaluated using the CCK kit. HCT116 cells in the logarithmic growth were inoculated to 96-well plates at a density of 5000 cells per well, cultured overnight, and different concentrations of nanoparticles were added into the culture medium and kept for 3 h. After the nanoparticles were internalized by the cells, the upper medium was taken out and the cells were washed with PBS three times to remove the undevoured nanoparticles. Then the cells were irradiated by an 808 nm laser at 1.5 W for 3 min. Finally, 10% CCK culture medium was added (808) at 100 µL/well, and the cells were incubated at 37 °C for 4 h. The OD value was detected by Microplate System at a detection wavelength of 450 nm. The cell viability was calculated according to the following equation:

$${\text{Cell activity value }}\left( \% \right) \, = \, \left( {{\text{OD}}_{{{\text{experimental}}}} - {\text{ OD}}_{{{\text{blank}}}} } \right)/\left( {{\text{ OD}}_{{{\text{control}}}} - {\text{ OD}}_{{{\text{blank}}}} } \right) \, \times { 1}00\%$$

### Wound-healing assays

Human Umbilical Vein Endothelial Cells (HUVECs) were seeded on 6-well plates at a density of 10^6^ cells per well. 5 ng/mL vascular endothelial growth factor (VEGF) was added to experimental groups. The migration of the cells was monitored on a Leica inverted fluorescence microscope DM3000 after the cells were treated for 24 h with 0.1 mg/mL Zn_0.4_Fe_2.6_O_4_ and AFZDA, respectively. The migration rate of the HUVECs was calculated based on the control group.

### Tube formation assays

HUVECs were seeded on 10 μL Matrigel-coated (corning) μ-Slide Angiogenesis (ibidi) and formed tubes. 10^4^ cells were seeded and treated with 0.1 mg/mL of Zn_0.4_Fe_2.6_O_4_ and AFZDA, respectively. 40 ng/mL vascular endothelial growth factor (VEGF) was added to the experimental groups. Then the cells were cultured at 37 °C with 5% CO_2_ and were observed every 30 min for 24 h. Photographs were acquired on a Leica inverted fluorescence microscope DM3000.

### Animal experiment

Human colon carcinoma HCT116 cells better mimic therapeutic effects in vitro, and CT26 mouse colon cancer cells are used to establish a syngeneic mouse model for exploring the immune response in therapy. Five-week-old BALB/c mice and BALB/c nude mice (for colorectal studies) were purchased from the experimental animal center of Shanghai Jiao Tong University. The animal experiments were carried out according to the ethical guidelines of Shanghai Jiao Tong University. 20 μL of PBS containing 2 × 10^6^ CT26 cells was injected into the right front leg of each BALB/c mice for building a tumor model. The tumor was allowed to grow to the size of 100 mm^3^ before in vivo experiments.

The tumor-bearing mice were injected via tail vein with 0.5 mg/kg AFZ, AFZD, or AFZDA NPs once every other day. Tumor volume (V) was calculated by equation: V = width^2^ × length/2. The mice in each group were anesthetized and killed, and the tumors were harvested and dissected. And mouse body weights were recorded after 24 days of treatment.

### Histological image

The tumor and tissues of mice were removed and fixed with 4% poly formalin for 72 h and embedded in paraffin and were then dehydrated with gradient alcohol. These tissues were cut into 4 μm-thick slices with a microtome. Next, the antigen was repaired by microwave for 20 min and then blocked by endogenous peroxidase 30% H_2_O_2_ for 30 min. Then 3% BSA was added, incubated for the 30 s in darkness, and immunofluorescent staining was conducted for imaging using a confocal laser scanning microscope.

### In vivo fluorescence imaging

Mice-bearing CT26 tumor was injected intravenous with nanoparticles (2.0 mg/kg). The fluorescent images were recorded after injection 2, 4, and 24 h. The main organs were harvested from the mice treated with AFZD and AFZDA NPs for 24 h. The quantitative fluorescence signals were analyzed for mice and major organs (n = 3).

### PA and MR imaging

Mice-bearing CT26 tumors were treated with saline and AFZDA nanoparticles (0.5 mg/kg) by intravenous injection. At 30 min after injection, PA and MR photographs were taken to evaluate the in vivo tumor imaging ability of the AFZDA nanoparticles. Subcutaneously, the PA imaging of AFZDA nanoparticles was observed through a photoacoustic microscope. The T1-weighted MRI of tumor-bearing mice was also obtained. The AFZDA NPs with various concentrations were dispersed in saline for in vitro experiments and the images were collected.

## Results and discussion

The detailed procedure for preparing AFZDA NPs is illustrated in Fig. 1a. AFZ NPs were prepared by solvothermal in-situ synthesis. DOX and anti-ANG2 were loaded via physical adsorption in mesoporous channels of the AFZ NPs. In this work, the normalization of the vessels could increase the ability of delivering oxygen and immune cells to the tumor by reconstructing the intratumoral environment, as shown in Fig. 1b. AFZDA NPs could promote the close connection of endothelial cells with perithelial cells by regulating VEGF and ANG2, normalizing the structure and function of vessels. The hypoxia in the tumor was thus alleviated, and the infiltration of drugs and anti-tumor immune cells in the tumor was promoted. The thermo-chemotherapy effects were improved through vascular normalization of the tumor, as shown in Fig. 1c.

**Fig. 2:**
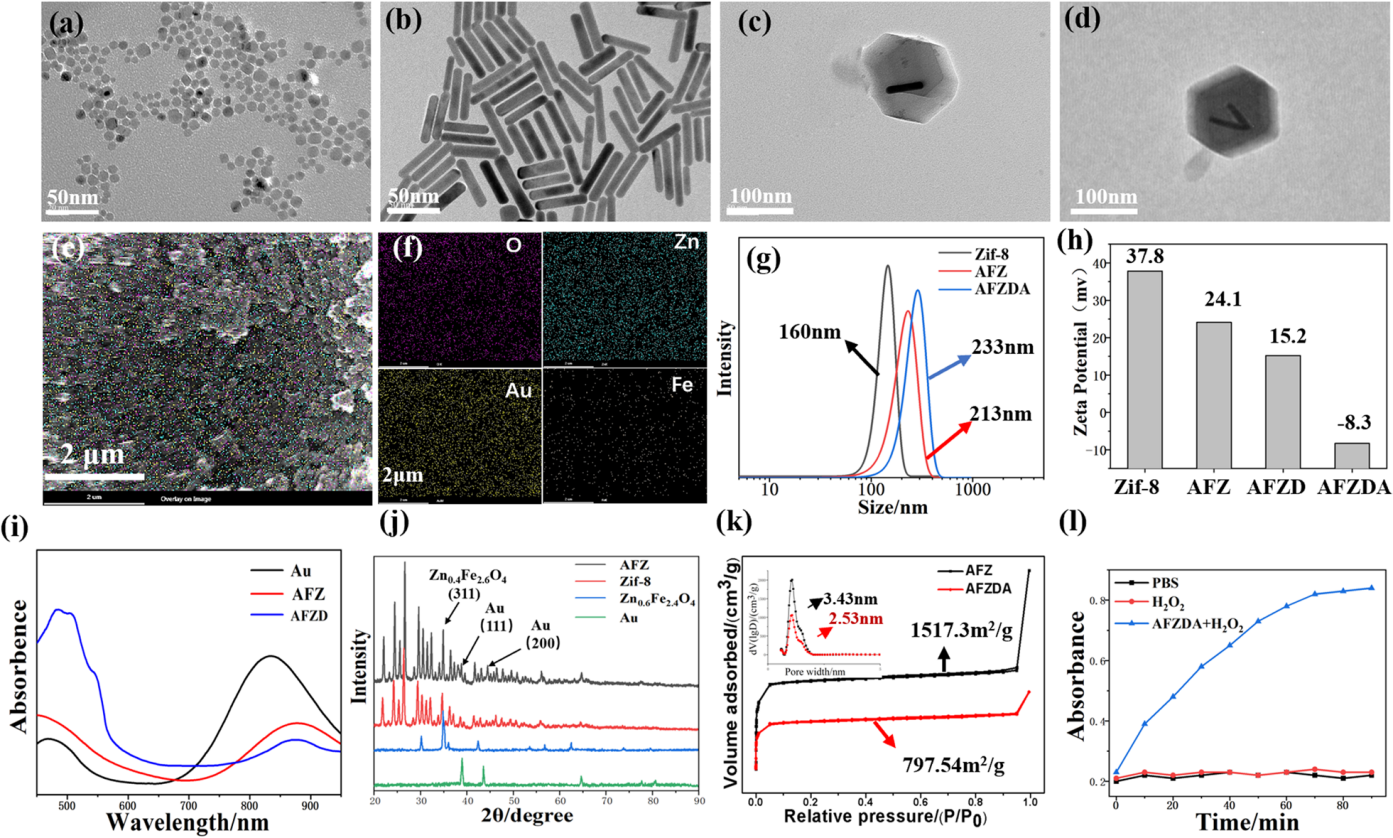
TEM images of Zn_0.4_Fe_2.6_O_4_ NPs **a**, Au nanorods **b**, AFZ **c**, and AFZD **d** and SEM image with element mapping **e**–**f** of AFZ; size distribution based on DLS **g** and Zeta potential **h** of different samples; UV-vis-NIR of AFZ NPs and Au nanorods **i**; XRD **j** of Zif-8 and AFZ; The sorption and desorption curves and pore size distribution of AFZ and AFZDA (**k**). **l** Time-dependent TMB consumption with AFZDA NPs

TEM imaging was used to analyze the structure of Zn_0.4_Fe_2.6_O_4_ NPs, Au nanorods, and AFZ, as shown in Fig. 2a, b, and c, respectively. Zn_0.4_Fe_2.6_O_4_ NPs with an average size of ca. 6 nm were prepared with a hydrothermal method (Fig. 2a). The elementary composition of Zn_0.4_Fe_2.6_O_4_ nanoparticles was characterized by EDX, which shows the quantified ratio between zinc, iron, and oxygen is 0.39 to 2.61 to 0.4. The Au nanorods that were prepared with a seedless method had clear rod-like profiles (Fig. 2b). In Fig. 2c, Au nanorods and Zn_0.4_Fe_2.6_O_4_ NPs were encapsulated in Zif-8 (AFZ). As shown in Fig. 2d, DOX loading was achieved by absorbing DOX molecules in channels and surface of the AFZ due to the good physical adsorption properties. SEM imaging was adopted to characterize the surface morphology and element distribution of AFZ NPs, the results of which are shown in Fig. 2e–f. The AFZ NPs exhibited a narrow size distribution and uniform distribution of O, Zn, Au, and Fe elements. The size distribution of different samples was measured based on dynamic light scattering (DLS) (Fig. 2g). The average size increased from 160 nm (Zif-8) to 213 nm (AFZ) due to the incorporation of Au nanorods and Zn_0.4_Fe_2.6_O_4_ NPs, which was consistent with the results of TEM imaging. The average size of AFZDA was 233 nm, ascribed to the encapsulation of DOX and adsorption of electronegative anti-ANG2. In addition, zeta potential values of different nanoparticles are shown in Fig. 2h. The incorporation of Zn_0.4_Fe_2.6_O_4_ NPs, Au nanorods, and DOX decreased the zeta potential of the Zif-8 particles. The negatively charged anti-ANG2 greatly decreased the zeta potential when it was adsorbed onto the surface of the AFZDA NPs, from 15.2 to −8.3 eV.

As shown in Fig. 2i, the absorption spectra of AFZD NPs, AFZ NPs, and Au nanorods in their aqueous solutions were obtained by UV–vis-NIR spectroscopy. The wide absorption band of AFZD NPs in the NIR region was close to that typical of Au nanorods, showing their good photothermal conversion under 808 nm laser irradiation.

Furthermore, the XRD patterns of Au, Zn_0.4_Fe_2.6_O_4_ NPs, Zif-8, and AFZ NPs are shown in Fig. 2j. Zif-8 had a rhombic dodecahedron structure, whose typical diffraction peaks were also found in the pattern of AFZ NPs. Moreover, the peaks at 38.1° and 44.3° that are assigned to (111) and (200) planes of Au nanorods and the peak at 35.8° that is assigned to (311) plane of Zn_0.4_Fe_2.6_O_4_ NPs appeared in the pattern of AFZ NPs. These results indicate that Zn_0.4_Fe_2.6_O_4_ NPs and Au nanorods were incorporated into the Zif-8. To assess the drug loading capacity of AFZ, the nitrogen adsorption by AFZ and AFZDA NPs was measured by BET and the curves displayed a type I isotherm, as shown in Fig. 2k. The BET surface area decreased from 1517.3 m^2^/g to 797.54 m^2^/g, and the average diameter of micropores decreased from 3.43 nm to 2.53 nm due to the DOX loading and the adsorption of anti-ANG-2 at the surface and in the porous channels of the AFZ (Fig. 2k). The loading capacity of DOX was determined as 143 mg/g, and the loading of anti-ANG-2 was about 11 mg/g. In the presence of H_2_O_2_, Zn_0.4_Fe_2.6_O_4_ nanoparticles were gradually released from AFZDA NPs due to collapsing of the frame structure. The colorimetric assays employing 3,3′,5,5′-tetramethyl-benzidine (TMB) were utilized to validate the peroxidase (POD)-like activity of Zn_0.4_Fe_2.6_O_4_ nanoparticles. The decrease of TMB concentration was observed with time (Fig. 2l).

**Fig. 3:**
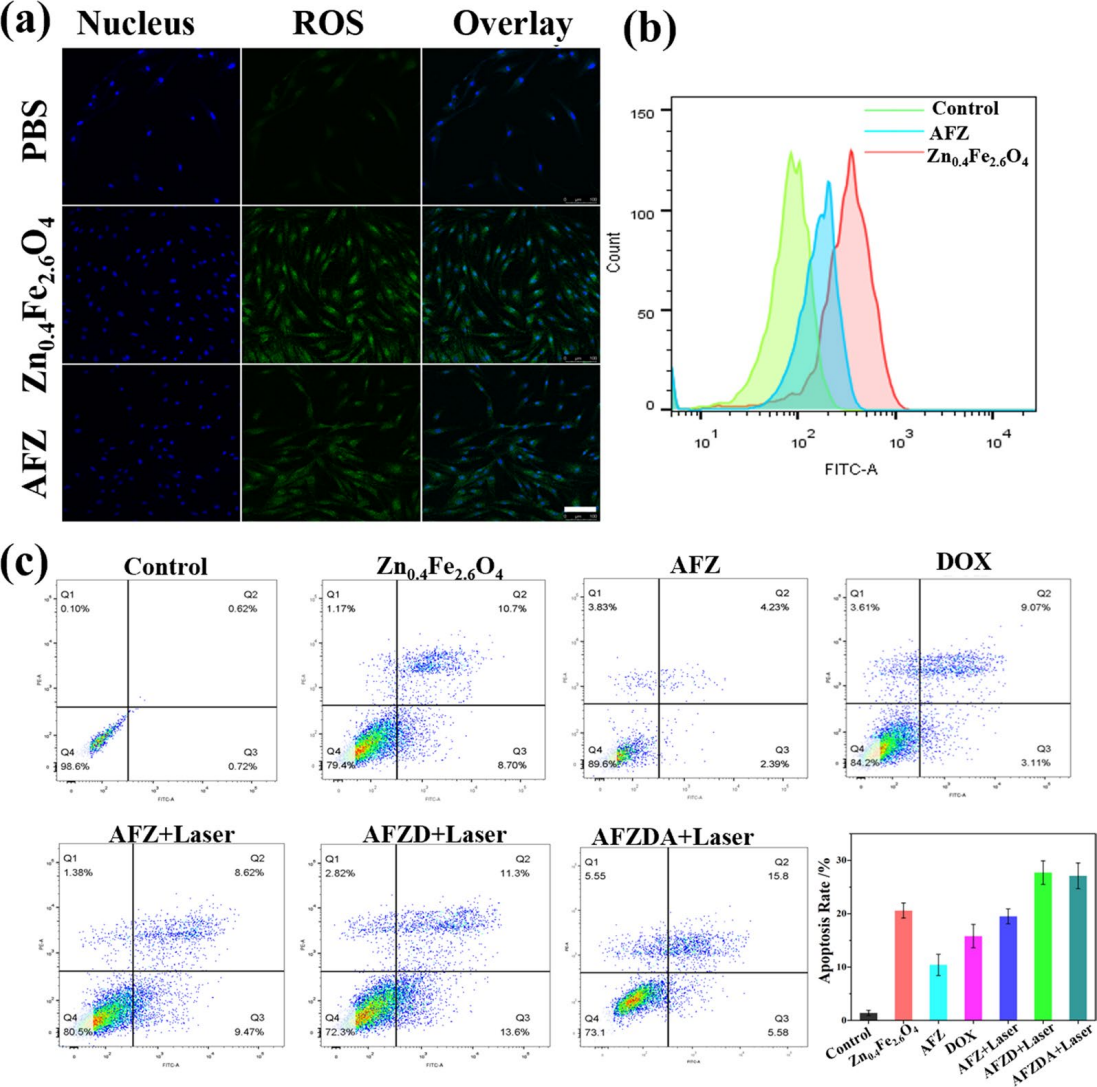
**a** CLSM imaging of intracellular ROS stained with DCFH-DA after different treatments and **b** Flow cytometry. Scale bar = 60 μm. **c** Cell apoptosis after various treatments was evaluated by flow cytometry (n = 4, mean ± s.d.)

The presence of intracellular ROS was imaged by laser scanning confocal microscopy, as shown in Fig. 3a. The ROS expression dramatically increased after treatment with Zn_0.4_Fe_2.6_O_4_ nanoparticles. Comparatively, the intracellular ROS was reduced when treated with AFZ nanoparticles, due to Zif-8 coating (Fig. 3b). Annexin-v/Propidium iodide (PI) double staining kit was used to evaluate the apoptosis of HCT116 cells with different treatments. The cell apoptosis was analyzed by flow cytometry, the results of which are shown in Fig. 3f. The apoptosis rate of the Zn_0.4_Fe_2.6_O_4_ -treated group was 20.57% due to reactive oxygen species (ROS). In comparison, the apoptosis rate of HCT116 cells treated with AFZ NPs was only about 10%, while the apoptosis rate of the AFZ, AFZD, and AFZDA NPs-treated group significantly increased under NIR laser irradiation (808 nm, 1.5 W/cm^2^, 3 min). These results indicated that the Zif-8 could reduce the effects of CDT and PTT, and the cell apoptosis induced CDT and PTT effect under the NIR irradiation and nanoparticles released with the increase of temperature. The release of DOX from the AFZD NPs was investigated.

**Fig. 4:**
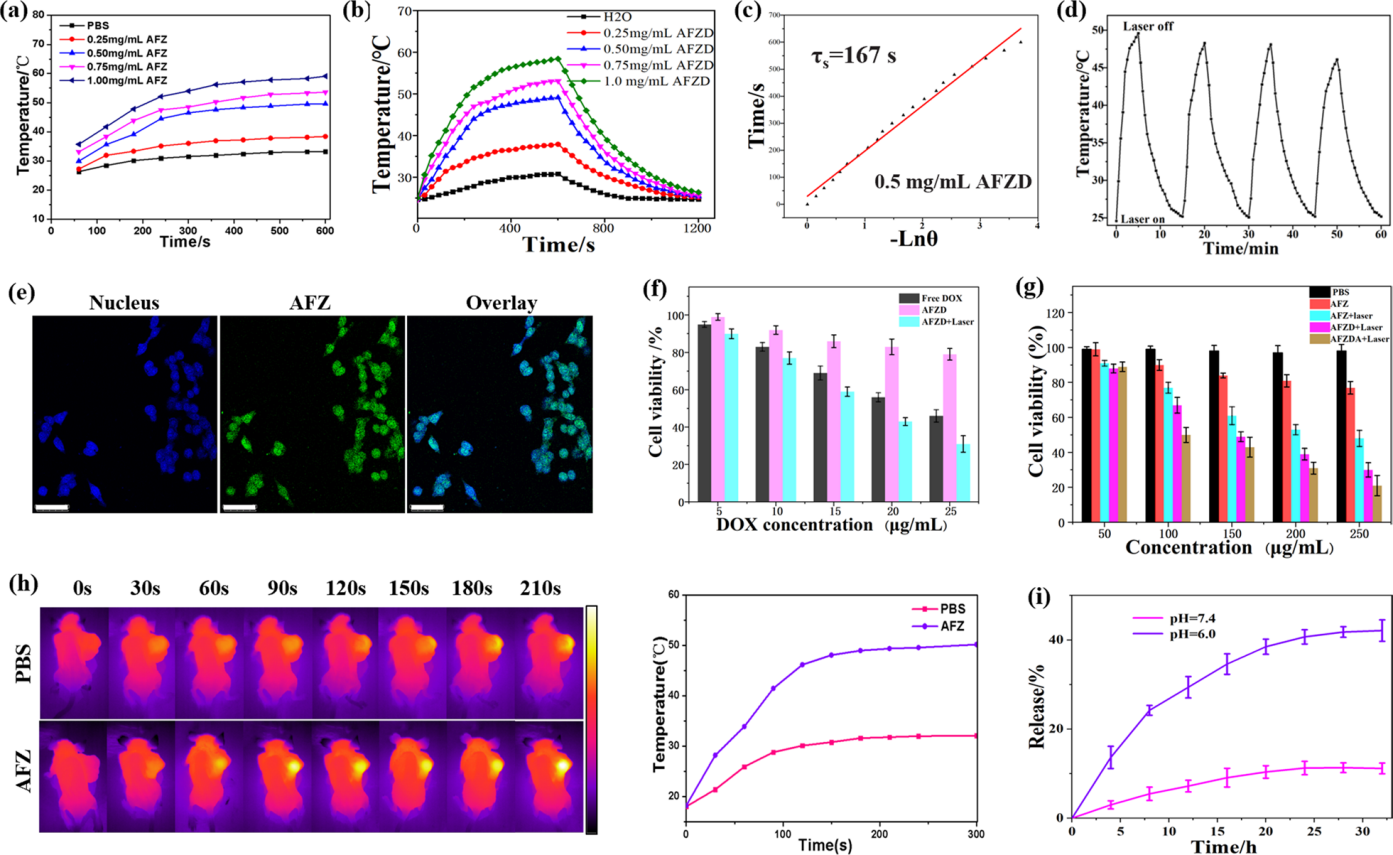
**a** Temperature variation curve of different concentrations of AFZD NPs (0.25 ~ 1.00 mg/mL) under the irradiation of a NIR laser (808 nm, 1.5 W/cm^2^) for 600 s. **b** Temperature elevation of the aqueous AFZD with different concentrations (0, 0.25, 0.5, 0.75, 1.0 mg/mL) under the irradiation of a NIR laser (808 nm, 1.5 W/cm^2^) for 600 s and shutting off the laser. **c** The determination of the time constant for heat transfer from the system using linear regression of the cooling profile of the AFZD with 0.5 mg/mL concentration. **d** Cyclic photothermal stability of AFZ NPs. **e** Laser scanning confocal microscopy images of HCT116 cells cultivated with AFZ NPs. Scale bar = 50 μm. **h** Infrared images of mice after administration of PBS buffer and AFZ NPs and the temperature changes with irradiation time (808 nm, 1.5 W/cm^2^). **f** Viability of HCT116 cells treated with the same DOX concentrations in AFZD NPs and free DOX (n = 4, mean ± s.d. p < 0.05). **g** Viability of HCT116 cells treated with different concentrations of AFZ, AFZD and AFZDA NPs (n = 4, mean ± s.d. p < 0.05) under different conditions. **i** Cumulative DOX release from AFZD NPs (0.75 mg/mL) at different pH values (n = 4, mean ± s.d.)

The temperature changes of the AFZD NPs and H_2_O with irradiation time were recorded, as shown in Fig. 4a. At each concentration, the temperature of AFZD solutions increased with time under irradiation. The temperature increase was more significant at higher concentrations, as expected. At the concentration of 0.5 mg/mL, the temperature of AFZD solution increased from 24.8 °C to 49.1 °C after the NIR laser irradiation for 600 s. In Fig. 4b, temperature elevation of the aqueous AFZD at different concentrations (0, 0.25, 0.5, 0.75, 1.0 mg/mL) under the irradiation of a NIR laser (808 nm, 1.5 W/cm^2^) for 600 s and shutting off the laser. As shown in Fig. 4c, τ_s_ was determined to be 167 s for the concentration of 0.5 mg/mL AFZD by linear fitting cooling time (t) to negative natural logarithm of temperature (−ln θ). η was ultimately calculated to be 18.1%. These results showed that AFZD solutions possessed significant photothermal conversion capability in vitro.

As shown in Fig. 4d, the photothermal stability of AFZ NPs (0.75 mg/mL) was characterized. The temperature of AFZ NPs solution increased from 24.8 ℃ to 49.8 ℃ after NIR laser irradiation for 5 min. When the irradiation was off, the temperature decreased to the original value after 6 min. Subjected to 4 on–off cycles, the AFZ NPs did not show fatigue signs, suggesting good photothermal stability.

The endocytosis of AFZ NPs was observed by laser scanning confocal microscopy (Leica Co.). The AFZ NPs labeled with FITC were co-incubated with HCT116 cells (the nuclei of which were stained with DAPI) for 3 h before the cells were fixed and stained (Fig. 4e). The LSCM images indicate that nanoparticles were internalized by the HCT116 cells, as evidenced by the overlap of green and blue (DAPI) fluorescence. Our previous studies have shown that the AFZ and AFZD NPs have excellent near-infrared absorption property. The AFZD NPs with different concentrations were irradiated by a near-infrared (NIR) laser (808 nm, 1.5 W/cm^2^).

As shown in Fig. 4f, the CCK-8 method was used to study the biocompatibility and cytotoxicity, the same DOX concentrations, AFZD NPs group has lower cytotoxicity than free DOX, but higher cell toxicity under irradiated (808 nm, 1.5 W/cm^2^). In Fig. 4g, the viability of HCT116 cells was treated with AFZ, AFZD, and AFZDA NPs in vitro to explore the biocompatibility and therapeutic effects of the nanomaterials. The cell viability of HCT 116 cells treated with the nanoparticles of different concentrations was compared. With increasing concentration of the nanoparticles, the viability of cells with different treatments decreased. At the same concentration, the cells treated with AFZ NPs without laser irradiation showed relatively high viability. When the laser irradiation was applied for 3 min, the cell viability decreased significantly. The cells treated with AFZD and AFZDA NPs under laser irradiation showed even lower viability since the loaded DOX and/or anti-ANG-2 exert therapeutic effects on the cancer cells. At the concentration of 250 μg/mL, the cell viability was lower than 20%. These results indicate the great cancer cell inhibition ability of the nanoparticles. Also, the photothermal therapeutic effect under irradiation was confirmed.

Tumor-bearing mice were subcutaneously injected with 2 mg/kg of AFZ NPs as well as PBS buffer as a control, and real-time infrared imaging was performed for the mice under NIR laser irradiation (808 nm, 1.5 W/cm^2^). The temperature of the tumor site was detected by an infrared thermal camera at every 30 s. As can be seen from the temperature curves in Fig. 4h, the temperature of the tumor in the mouse injected by AFZ NPs increased from 20.5 °C to 50.9 °C In comparison, the temperature of the tumor in the mouse injected with PBS buffer only increased to 33.1 °C. Figure 4i shows the DOX release curves of AFZD during a period of 24 h at pH = 7.4 and 6.0, respectively. At pH 7.4 typical of physiological environment, the cumulative release of DOX was only 22.6% at 24 h. It is characteristic of the endolysosomal system in cancer cells at pH 6.0, however, more than 90% of the loaded DOX was released. These results indicate that the nanoparticles kept stable under physiological conditions, which were able to release the chemo-therapeutic drug under intracellular conditions.

**Fig. 5:**
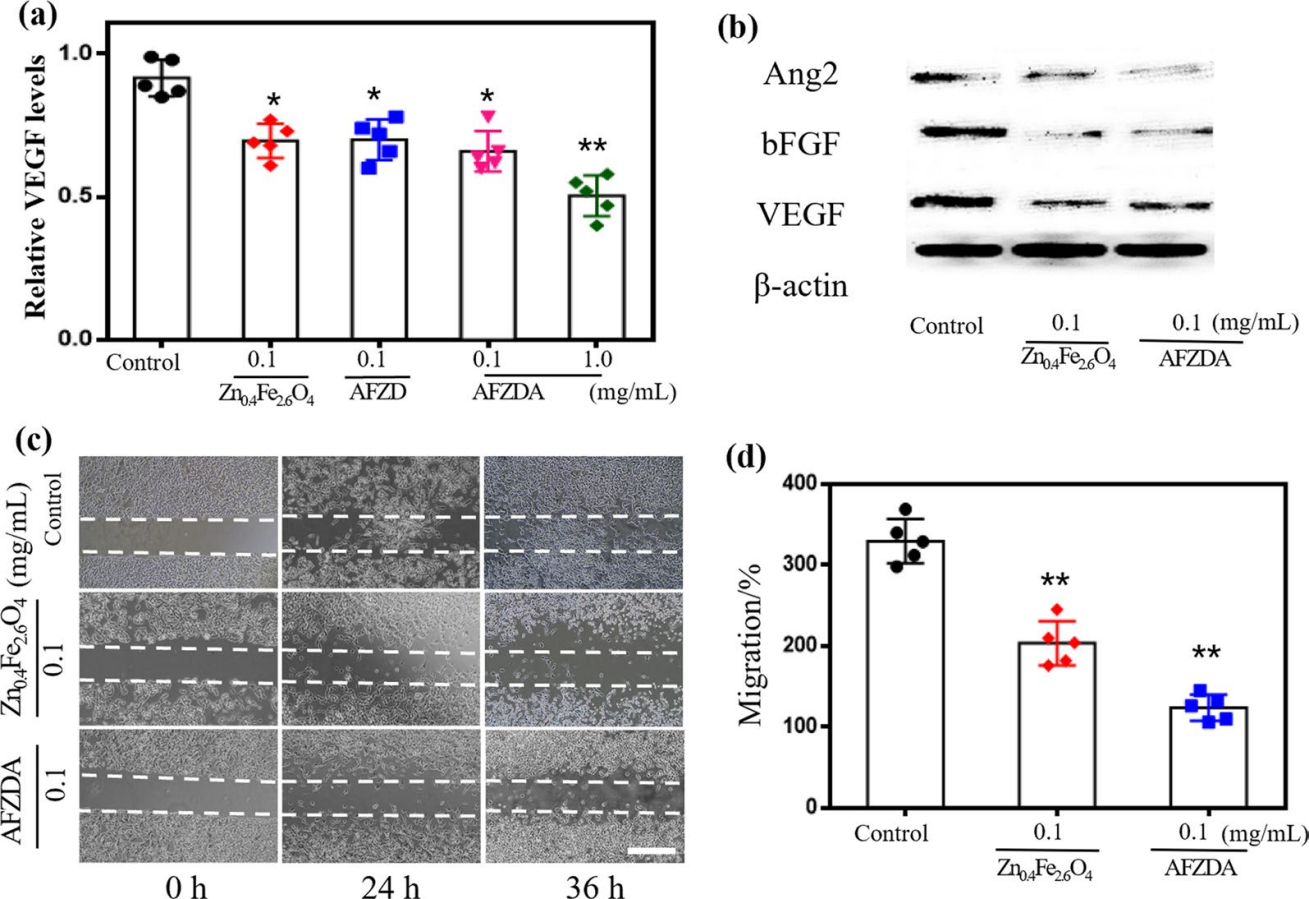
**a** Zn_0.4_Fe_2.6_O_4_ NPs, AFZD NPs, and AFZDA NPs inhibiting VEGF of HUVECs measured by Elisa kit Elisa at 24 h. **b** Western blot analysis for Ang2, b-FGF, and VEGFA expression in 0.1 mg/mL Zn_0.4_Fe_2.6_O_4_ NPs and AFZDA NPs treated HUVECs and β-actin was used as the internal reference. **c** The wound-healing assay indicating the migration ability of HUVECs using Vasculife-MV complete kit (0 h, 24 h, and 36 h after treatment with PBS or 0.1 mg/mL Zn_0.4_Fe_2.6_O_4_ NPs, scale bar = 200 µm. **d** The migration of HUVECs after treatment 36 h. All data are expressed as mean ± SD, n = 5 per group. P values were calculated using the two-paired Student’s t-test, treated groups vs. control group (*P < 0.05,**P < 0.01, ***P < 0.001)

The factors related to blood vessel normalization include VEGF, b-FGF, and Angiopioetin. A VEGF-MV Complete Kit was used to culture HUVECs, which were then treated with Zn_0.4_Fe_2.6_O_4_, AFZD NPs, and AFZDA NPs for 24 h, respectively. As shown in Fig. 5a, the expression level of VEGF decreased compared with the control group. When the HUVECs were treated with 0.1 mg/mL AFZDA NPs, the VEGF expression decreased by 35.7%. The western blotting results in Fig. 5b indicate that the Zn_0.4_Fe_2.6_O_4_ NPs reduced the secretion of VEGFR1 and b-FGF, and the AFZDA NPs could inhibit the expression of Angiopioetin2.

Wound healing experiments were performed to examine the effects of AFZDA NPs on cell migration. As shown in Fig. 5c, both Zn_0.4_Fe_2.6_O_4_ and AFZDA NPs hampered the migration of the HUVECs to the scratch zone. The AFZDA NPs inhibited the lateral transfer of the cells more significantly, reducing the migration rate by 61% compared to the control group.

**Fig. 6:**
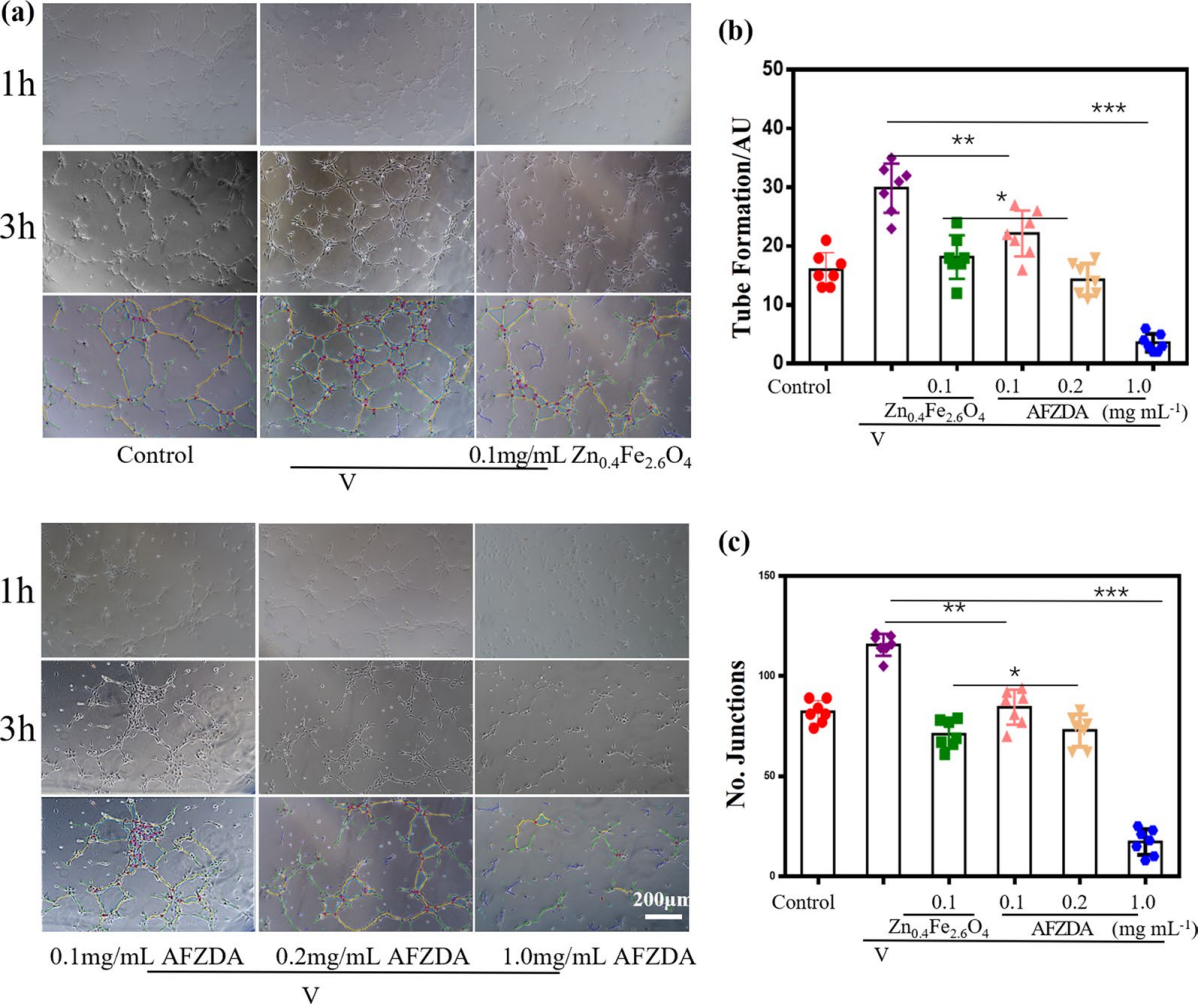
**a** Effects of Zn_0.4_Fe_2.6_O_4_ NPs and AFZDA NPs on tube formation of HUVECs induced by VEGF. HUVECs were inoculated on the Matrigel and treated with VEGF (40 ng/mL) in the presence or absence of either Zn_0.4_Fe_2.6_O_4_ NPs and AFZDA NPs (scale bar = 200 µm). The quantitative of tube formation **b** and junctions **c** were evaluated after different treatments by angiogenesis analysis from image J software. (n = 7, mean ± s.d.) *P < 0.05, **P < 0.01, ***P < 0.001 versus control

The effects of Zn_0.4_Fe_2.6_O_4_ and AFZDA NPs on the vessel formation ability of the vascular endothelial cells were investigated. The HUVECs were added to the 24-well plates with Matrigel and incubated overnight. Then different formulations were added to the wells, which were then incubated for 24 h. Then the HUVECs were seeded in Matrigel and cultured in the incubator for 0.5–6 h. A large amount of HUVECs contacted and fused to form a tube structure incubating for 1 h, which further formed a network structure incubating for 3 h. Thereafter the tubular structure disintegrated gradually. The dynamic process of the angiopoiesis was observed by microscopy, as shown in Fig. 6. The software Image J was used to analyze the number of tubes and nodes. In the case of treatment with 0.1 mg/mL Zn_0.4_Fe_2.6_O_4_ and AFZDA NPs, the number of tubes formed was 18 ± 1.4 and 3 ± 0.8, respectively. The number of tubes of the control V group was 31 ± 2.5 (Fig. 6b). Treated with 0.1 mg/mL AFZDA NPs, the number of nodes formed was 21 ± 4.8, compared to that of the control V group, 114 ± 5.8 (Fig. 6c). These results indicate that treatment with 0.1 mg/mL AFZDA NPs for 24 h was capable of inhibiting the tube formation of the HUVECs in vitro.

**Fig. 7:**
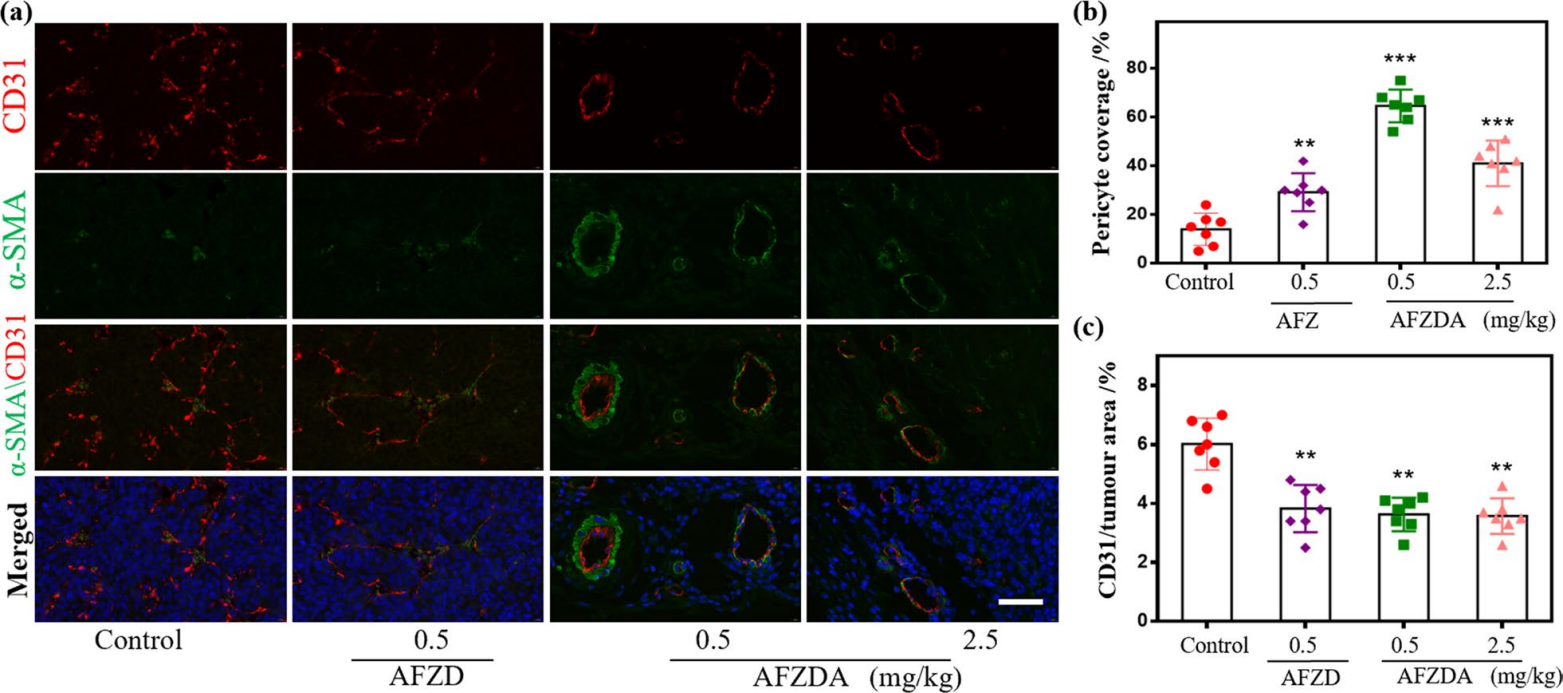
**a** The representative images and morphologic analysis of double staining of tumor sections for CD31 (endothelial cells, red), α-SMA (pericyte cells, green), and DAPI (nucleus, blue) after treatment with AFZD and AFZDA NPs at different doses ( scale bar = 50 µm); **b** pericyte coverage after nanoparticles treatment with different doses and **c** the CD31of tumor, (n = 7, mean ± s.d.); **P < 0.01, ***P < 0.001 versus control

To evaluate the ability of the AFZDA NPs to normalize the tumor vessel morphology, the maturity of the tumors in the mice treated with AFZD and AFZDA NPs was investigated. The tumor tissues were double stained with the tumor endothelial cell marker CD31 and perithelial cell marker α-SMA. Then the tumor-bearing mice were treated with AFZD and AFZDA NPs, respectively, every other day for 14 days. As shown in Fig. 7a, in the tumor tissues of the control group, a small amount of α-SMA positive cells dispersed around the HUVECs, and the vessel structure was immature. Besides, α-SMA positive cells existed in the CD31 positive regions. After treatment with 0.5 mg/kg AFZDA NPs, the α-SMA positive cells, and the CD31 positive cells aggregated densely and the coverage of perithelial cells was 76%, as shown in Fig. 7b. The vascular structure of the tumor became more intact. The number of blood vessels was reduced in comparison with the control group, as shown in Fig. 7c.

**Fig. 8:**
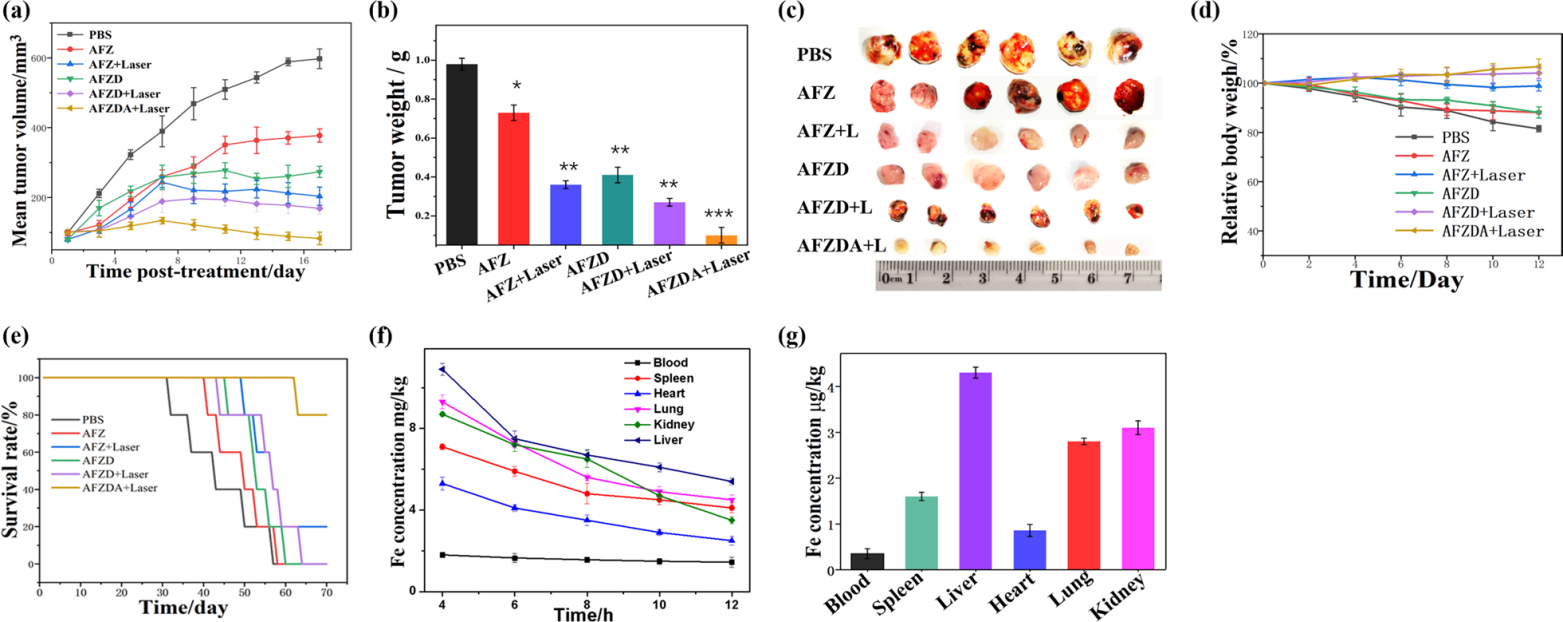
**a** Mean tumor volumes of the mice treated with different formulations at the nanoparticles dose of 0.5 mg/kg (808 nm laser irradiation, 1.5 W/cm^2^, 3 min); **b** Mean tumor weights. **c** The photograph of the tumors after different treatments and **d** The weight of tumor-bearing mice. **e** Survival curves from various treatments (nanoparticles dose 0.5 mg/kg). **f** Mean Fe concentrations (mg/kg) in organ with time after one single injection and **g** three consecutive injections. (n = 5, mean ± s.d.)

To further confirm the therapeutic effects of the nanoparticles, the tumor-bearing nude mouse model was built. The tumor-bearing mice were injected via tail vein with 0.5 mg/kg AFZ, AFZD, or AFZDA NPs once every other day. The nude mice in each group were anesthetized and killed, and the tumors were harvested, dissected, and observed after 24 days of treatment. As shown in Fig. 8a–d, the therapeutic effects of the nanoparticles on the tumor were consistent with the results of cell experiments discussed above. Figure 8a indicates the tumor growth inhibition effect of the nanoparticles, as the tumor growth of the mice treated with various formulations of nanoparticles was suppressed to different extents compared with that of the mice treated with PBS buffer. The hyperthermia effect of the AFZ NPs under NIR laser irradiation significantly inhibited the rapid growth of the tumor, but the subsequent tumor growth could not be controlled completely. When the hyperthermia effect was combined with DOX and anti-ANG2, the tumor growth inhibition became more significant, as reflected by the reduced volume of tumor after 8 days of treatment with AFZDA NPs. The tumor weight after treatment with different formulations was also recorded. As shown in Fig. 8b, the average tumor weight of the AFZDA NPs-treated mice group under laser irradiation was 0.1 g. In comparison, the average tumor weight of the AFZD NPs-treated group and that of the AFZ NPs-treated group under laser irradiation was 0.21 g and 0.73 g, respectively. These results were in good accordance with those of tumor volume. Figure 8c shows the photographs of the harvested tumors from the groups with different treatments, which further confirmed the therapeutic effects of the nanoparticles. The weight of the group treated with AFZDA NPs-treated group under laser irradiation had a slight increase, which indicated that AFZDA NPs have higher security, as shown in Fig. 8d. The survival time of tumor-bearing mice treated with different formulations was investigated, as shown in Fig. 8e. The mice treated with AFZDA NPs under irradiation had the highest survival rate, and no mice died within 60 days. The AFZDA NPs exhibited long-term anticancer activity, and the lifetime of tumor-bearing mice was effectively extended. The high anticancer efficacy of AFZDA NPs suggested the critical role of the normalization of tumor blood vessels in tumor treatment.

To investigate the body safety of AFZDA NPs, the distribution of element Fe in the mice body was studied. 0.5 mg/kg AFZDA NPs were injected into the mice via tail vein, and the Fe content in various organs of mice was measured, as shown in Fig. 8f. The amount of Fe ions in the organs gradually decreased with time after injection. The AFZDA NPs mainly accumulated in the liver, kidney, and lung. Then the AFZDA NPs were consecutively injected into the mice three times, and the Fe concentration in the organs is shown in Fig. 8g. The AFZDA NPs still preferentially accumulated in liver, kidney, and lung in vivo, indicating that the AFZDA NPs were metabolized primarily in liver and kidney.

**Fig. 9:**
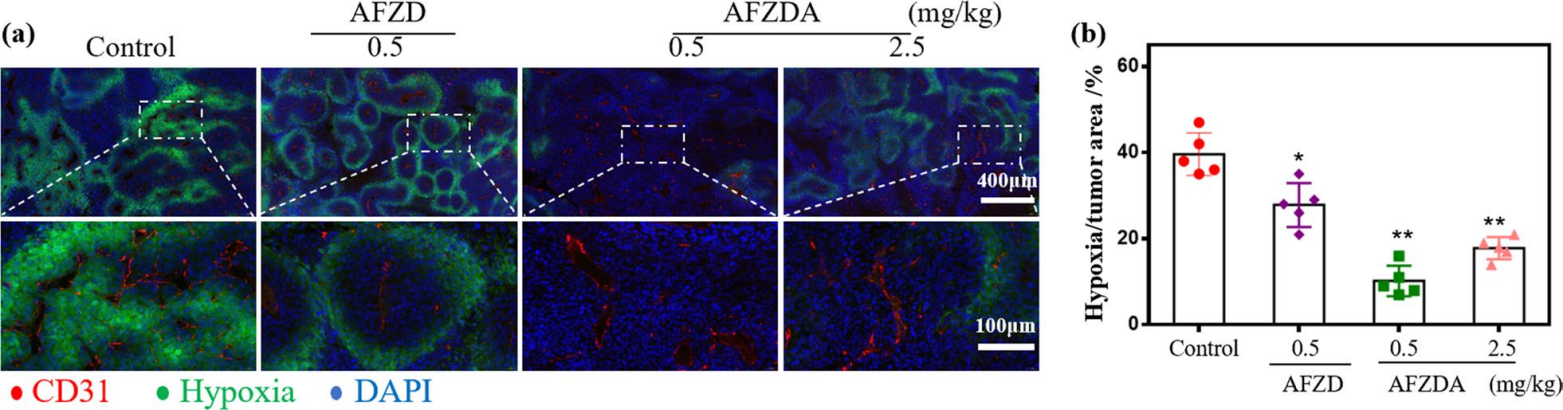
**a** Representative fluorescence images of CT26 subcutaneous tumor immunostained with CD31(endothelial cells, red), pimonidazole (hypoxia, green), and DAPI (nucleus, blue) staining. after treatment with AFZD and AFZDA NPs at different doses. **b** Quantification of the hypoxic area of tumor area following pimonidazole (50 mg/kg) injection and staining. (n = 5, mean ± s.d.); *P < 0.05, **P < 0.01 versus control

Hypoxia-inducing factor (HIF-1α) is highly expressed in tumor tissues due to hypoxia (Fig. 9). The green fluorescence (HIF-1α) was significantly reduced (AFZDA NPs group). Furthermore, vascular normalization helps to relieve hypoxia in tumor tissues through AFZDA NPs treated. The results again showed that the tumor hypoxia was improved because of AFZDA NPs. Remodeling of tumor immune microenvironment is a new direction of tumor therapy synergistically.

**Fig. 10:**
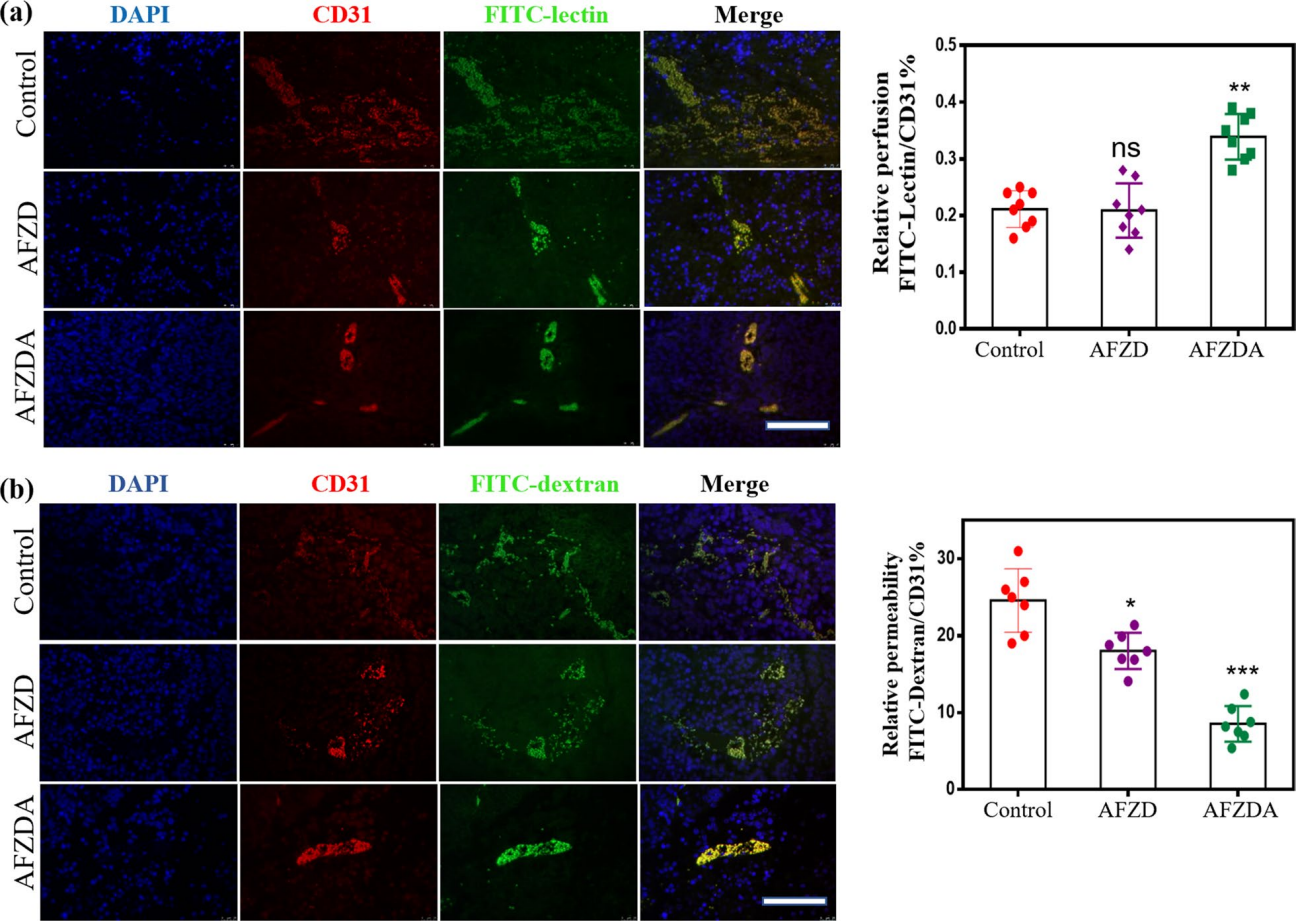
**a** The perfusion of tumor vessels measured by injection of FITC-lectin (green; red = CD31) after different treatments; **b** The vessel permeability observed by labeling with FITC-dextran (scale bar = 100 µm). The permeability of tumor vessels was measured. (n = 7, mean ± s.d.); Represents no significant difference (ns), *P < 0.05, **P < 0.01, **P < 0.001 versus control

As an adequate blood supply is required for tumor growth, local invasion, and metastasis, tumor blood vessels play an important role in the development of tumors. The morphology and structure of microvessels in tumor tissues are of great significance for understanding the treatment of tumors (Fig. 10a–b). The tumor-bearing mice were treated with AFZ and AFZDA NPs once every 2 days. FITC-lectin (green) was injected intravenously to mark the tissue vessels and CD31 (red) was used to label vascular endothelial cells. ANG2 in AFZDA inhibited angiogenesis and increased vascular perfusion. It can be seen from Fig. 10a that the vascular perfusion and diameter increased after the treatment with AFZDA NPs treatment. This indicated that the tumor blood vessels of mice treated with AFZDA NPs had significant changes in morphology and structure, which are signals of tumor vessel normalization. High interstitial pressure leads to drug penetration obstacles in tumor tissues [42]. The abnormal blood vessels make it difficult for nanoparticles to diffuse into tumor tissues under high interstitial pressure due to the proliferation of tumor cells and collagen interentanglement. In this work, dextran labeled with fluorescein isothiocyanate (FITC-dextran) and CD31 were used as vascular markers to observe the amount of dextran infiltrating into tumor tissues from blood vessels, which represents the permeability of blood vessels. After FITC-dextran (green) was injected into the mice's body, a part of the dextran remained in the vascular lumen and was attached to the surface of endothelial cells. There is a positive correlation between the density of blood vessels and the amount of dextran remaining in blood vessels in tumor tissues. The microvessel permeability could be expressed by the ratio of fluorescence intensity of FITC-dextran to that of CD31. The ratio of the AFZDA NPs-treated group was lower compared to the PBS group, indicating that the treatment with AFZDA NPs improved the tumor microenvironment and normalized the blood vessels, thus decreasing blood permeability. The mechanism is that the gradual degradation of collagen during the treatment decreases the tumor pressure and normalizes blood vessels, which facilitates photothermal tumor therapy.

**Fig. 11:**
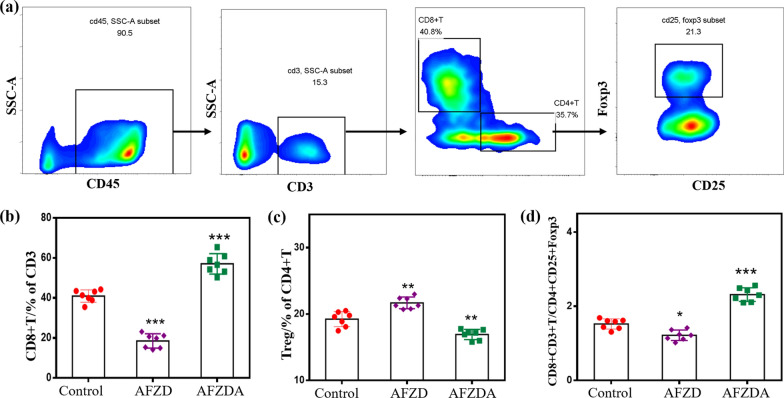
**a** Flow cytometric analysis of different groups tumors after treatment PBS, AFZD, AFZDA under laser (808 nm, 1.5 W/cm^2^, 3 min). Quantification of the data from the experiment and the percentage of CD8^+^T of CD3^+^T cells **b**, the percentage of Treg of CD4^+^T cells **c**, the percentage of CD8^+^ CD3^+^T of CD4^+^ CD25^+^Foxp3 cells (CTL/ Treg) **d**. (n = 7, mean ± s.d. *P < 0.05, **P < 0.01, **P < 0.001 versus control)

Immune T cells were analyzed to elucidate how they played the therapeutic role by autoimmune mechanism during the treatment. Furthermore, cytotoxic T cells ( CTL, CD8^+^ CD3^+^T cells) are the main effector cells of anti-tumor immunotherapy and can actively induce tumor-specific antigens. Flow cytometric analysis is shown in Fig. 11a. The number of CD8^+^T cells significantly increased in tumor tissues of the mice treated with AFZDA NPs, accounting for 53.6% of the total CD3^+^T cells (Fig. 11b). Although AFZD NPs had a good photothermal-chemo-therapeutic effect, the expression of immune effector cells was inhibited at the tumor sites.

Tregs can inhibit the immune response as a subset of CD4^+^T cells. As shown in Fig. 11c, the expression level of Treg was 19.24%, 21.68%, and 16.93% in tumors treated with PBS, AFZD NPs, and AFZDA NPs, respectively. These results showed that the differentiation of immunosuppressive cells was effectively inhibited by reversing the tumor hypoxia. The AFZDA NPs improved the tumor infiltration of CD8^+^T cells through blood vessel normalization and reduced the differentiation of the Tregs (CD4^+^CD25^+^Foxp3) to some extent. Thus, the ratio of CD8^+^T cells to Tregs increased, as shown in Fig. 11d.

**Fig. 12:**
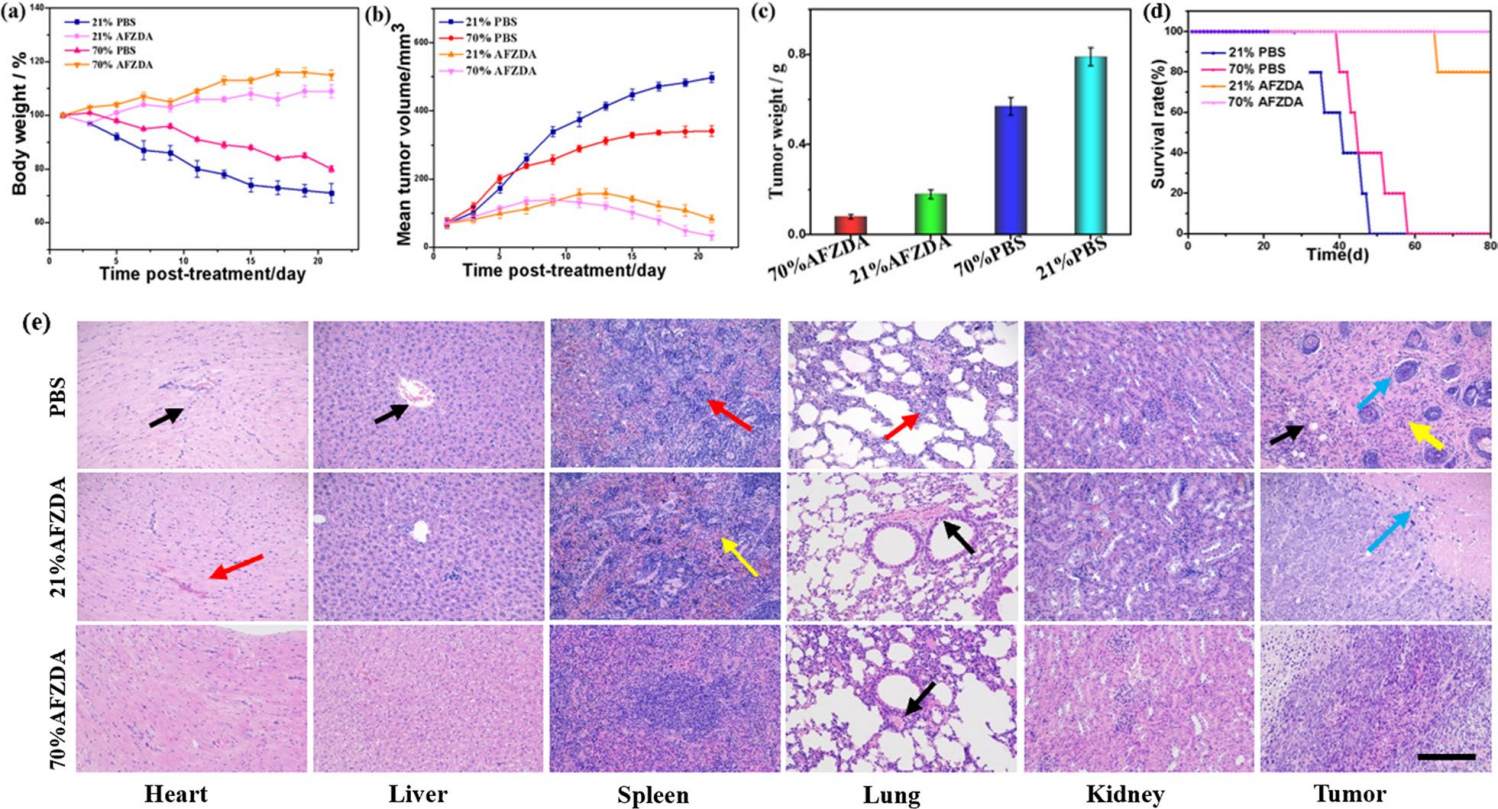
**a** Mean weight of tumor-bearing mice from respiratory different concentrations of oxygen after treatment PBS, AFZDA. **b** Mean tumor volume of different groups; **c** mean tumor weights (n = 4, mean ± s.d. P < 0.05) and **d** survival curves (AFZDA NPs dose 0.5 mg/kg) (n = 5). **e** Hematoxylin–eosin stained images of tissues and organs obtained. Scale = 200 μm

The oxygen concentration was further studied during tumor photothermal/ chemo-therapy treatment. After 21 days of treatment, the mice were anesthetized and killed, and the antitumor effects of different treatment groups were measured. The body weight, tumor weight, and tumor volume of mice in each group were compared, as shown in Fig. 12a–c. It was found that hyperoxia could be used as a treatment to inhibit tumor growth and slow down the tumor growth rate in mice. The body weight of the control group decreased by nearly 30% in 21 days, while only the high-oxygen treatment group decreased by about 20%. No apparent weight loss was observed in the respiratory 70% oxygen AFZDA treatment group(Fig. 12a). The tumor volume and weight, compared with the control group, were not only quickly inhibited, but the original tumor was shrinking. The final average tumor weight was less than 0.1 g (Fig. 12c), indicating that the AFZDA hyperoxia group had an obvious anti-tumor effect. The survival time of mice in each group treated by different methods was analyzed experimentally as shown in Fig. 12d. No death of mice occurred within 80 days in the AFZDA hyperoxia group. AFZDA NPs showed long-term anticancer activity. It was proved that the hyperoxia obviously inhibited cancer combined with photothermal/chemo-therapy and effectively extended the survival time of tumor-bearing mice.

The organs of mice and tumor tissues were stained by H&E, as shown in Fig. 12e. The toxicity was further studied during treatment in vivo. Compared with the control group, the treatment group of tissues and organs, no obvious inflammation and necrosis occurred. The structure of the myocardium was normal, and no obvious degeneration was found. There is hyperemia in the stroma, as shown by the red arrow. In the PBS group, obvious inflammatory cells were filtrated in the tissue, as shown in the black arrow. A small number of hepatocytes in the control group showed slight edema (yellow arrows). The overall structure of the spleen was normal, with connective tissue hyperplasia (yellow arrow). A large number of granulocyte infiltrates were seen in the control group (red arrows). No alveolar fusion and expansion, but the control group showed significant thickening (red arrow). The epithelial cell structure was full in the renal tissue and the glomerular structure was visible (as shown in the black arrow). The blue arrow showed tumor cells. A large number of inflammatory cells were observed in the PBS and AFZD groups (yellow arrows). The black arrows indicate that the inflammatory cells infiltrated the tumor tissue. The results of H&E staining showed that AFZDA NPs had no side effects on animals during treatment.

**Fig. 13:**
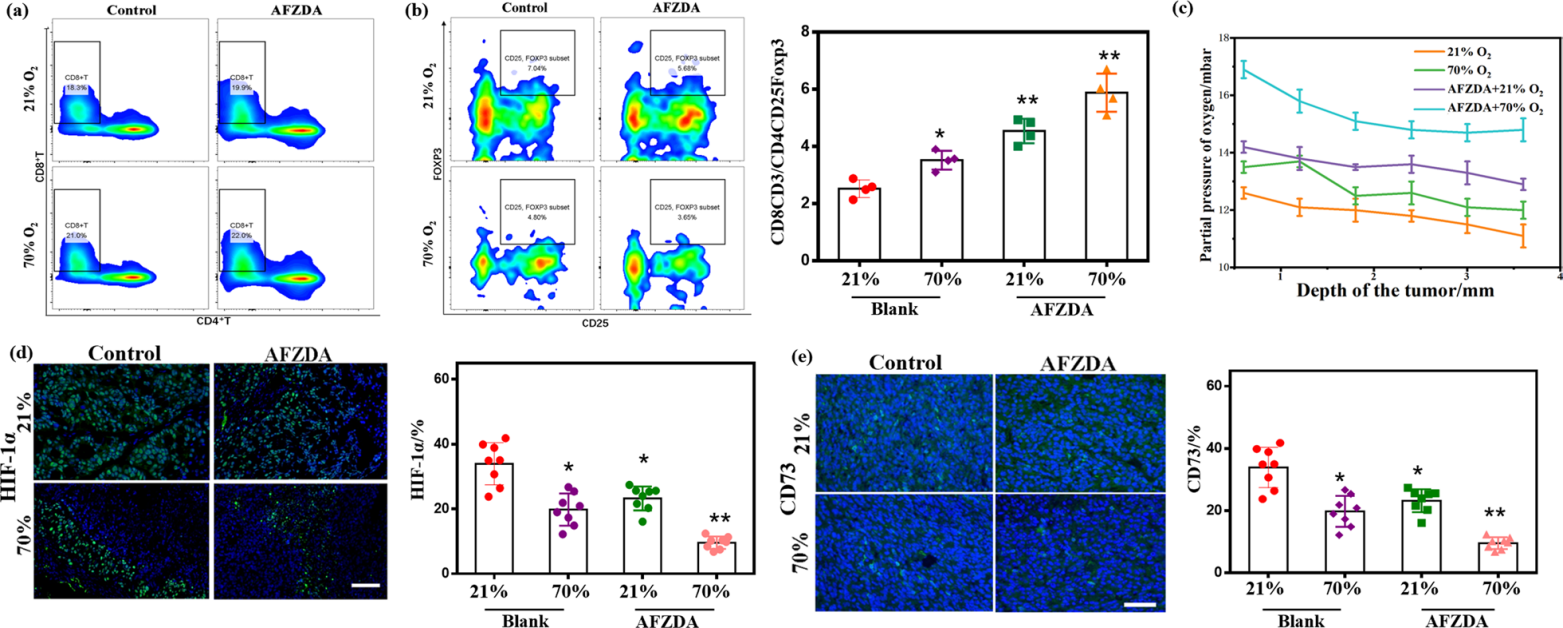
Flow cytometric analysis of the spleen from different treatment mice, the percentage of CD8^+^T cells of CD3^+^T cells **a** and Treg of CD4^+^T cells **b** (n = 5, mean ± s.d.). **c** The partial pressure of oxygen in tumors with the depth of the tumor, (n = 5, mean ± s.d.). The fluorescence imaging of HIF-1α **d** and CD73 **e** in vivo. Scale bar = 200 μm; quantification of HIF-1α and CD73 in tumors. (n = 7, mean ± s.d.); **P < 0.01, *P < 0.05 versus control

CD8^+^T cells are one of the main effector cells of anti-tumor immunotherapy and can actively induce tumor-specific antigens. The tumor-burdened Balb/c mice respectively were treated at 21% and 70% oxygen concentrations for 4 h/day for 4 days under NIR laser irradiation (808 nm, 1.5 W/cm^2^, 3 min). Flow cytometric analysis was shown in Fig. 13a. The proportion of CD8^+^T cells of spleen tissue was 18.3% in blank group mice, while 19.9% in AFZDA treatment group under 21% oxygen concentration. The proportion of CD8^+^T cells in the group treated with 70% oxygen was higher than that in the 21% oxygen group (22% and 21%, respectively). The results have shown that high oxygen concentration contributes to the differentiation of CD8^+^T cells in vivo. Tregs can inhibit the immune response as a subset of CD4^+^T cells. As shown in Fig. 13b, the expression level of Treg was 7.04% and 5.68% in tumors treated with PBS and AFZDA groups, respectively. When under 70% Oxygen, Treg expression was down-regulated to 4.8% in the PBS group and 3.65% in AFZDA group, respectively. The result showed that it effectively inhibited the differentiation of immunosuppressive cells by respiratory hyperoxia.

Breathing a high concentration of oxygen improves oxygen partial pressure (Fig. 13c). Besides, the hypoxia-inducing factor (HIF-1α) is highly expressed in tumor tissues due to hypoxia (Fig. 13d). After 70% oxygen treatment, the green fluorescence (HIF-1α) was significantly reduced. Furthermore, vascular normalization helps to relieve hypoxia in tumor tissues through AFZDA NPs treated. As we all know, the cells are metabolized mainly by glycolysis, leading to immunosuppression in the absence of oxygen. Hyperoxia therapy can inhibit tumor hypoxia and reduce intracellular adenosine formation. The expression of CD73 (in Fig. 13e) was blocked by the change in metabolism. The results again showed that the tumor hypoxia was improved because of AFZDA NPs. Remodeling of tumor immune microenvironment is a new direction of tumor therapy synergistically.

**Fig. 14:**
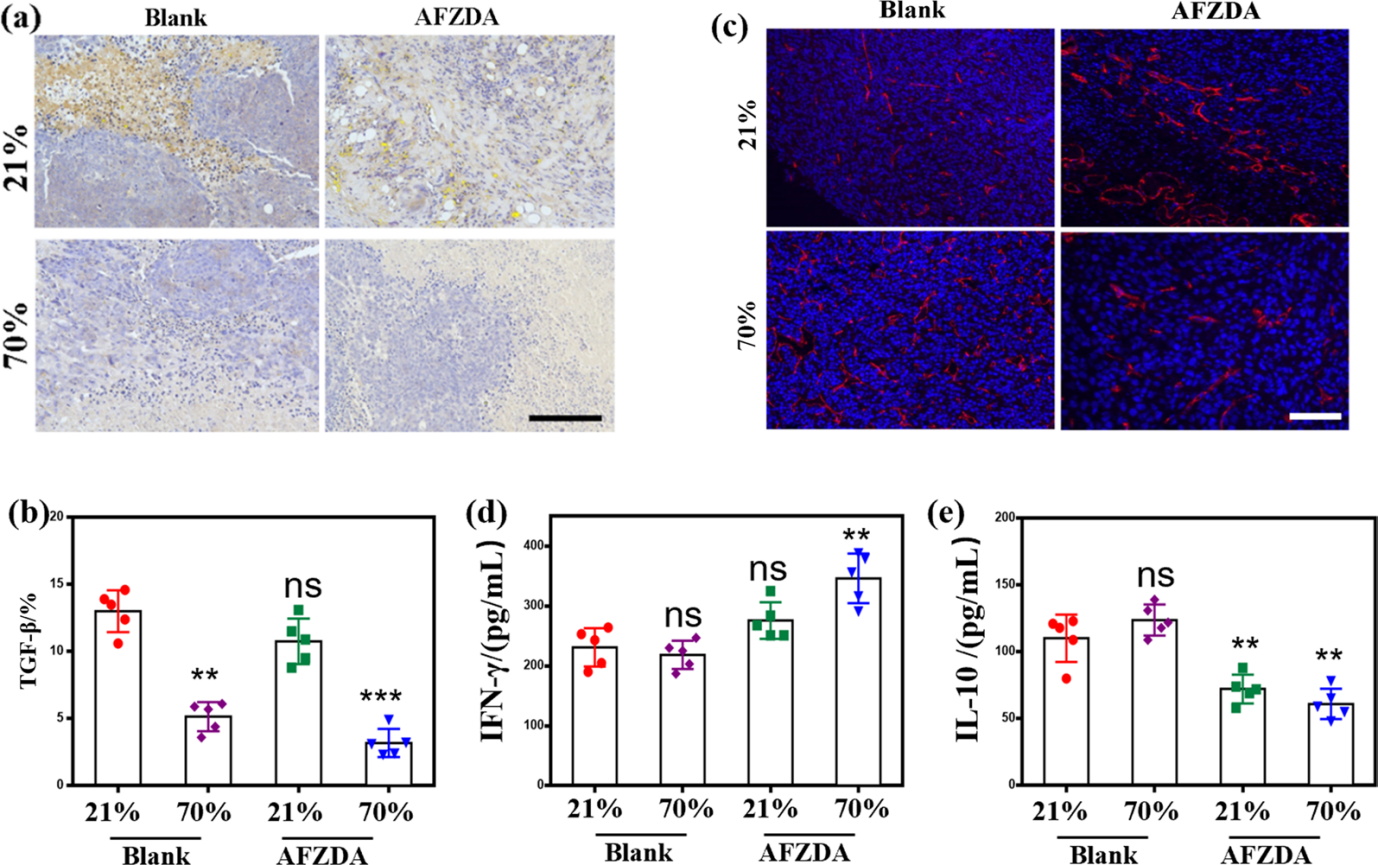
**a** TGF-β immunohistochemistry of tumor tissue, Scale = 200 μm. **b** Quantification of TGF-β in tumors. **c** TGF-β, **d** IFN-γ, and **c** IL-10 contents in tumors were tested using flow cytometry. (n = 5, mean ± s.d.); represents no significant difference (ns); **P < 0.01, ***P < 0.001, versus control

In terms of immune cells, the AFZDA + 70%O_2_ group showed significant differences with AFZDA treatment. Meanwhile, in terms of cytokines, the experiment further characterized the changes in tumor immune microenvironment during AFZDA treatment with a high concentration of oxygen. Compared with AFZDA, AFZDA + 70%O_2_ could not only reduce anti-inflammatory cytokines (TGF-β and IL-10) more effectively but also increase the level of anti-tumor pro-inflammatory cytokines IFN-γ in tumors (Fig. 14). The combination of the AFZDA and high- concentration oxygen had the strongest anti-tumor immune enhancement.

**Fig. 15:**
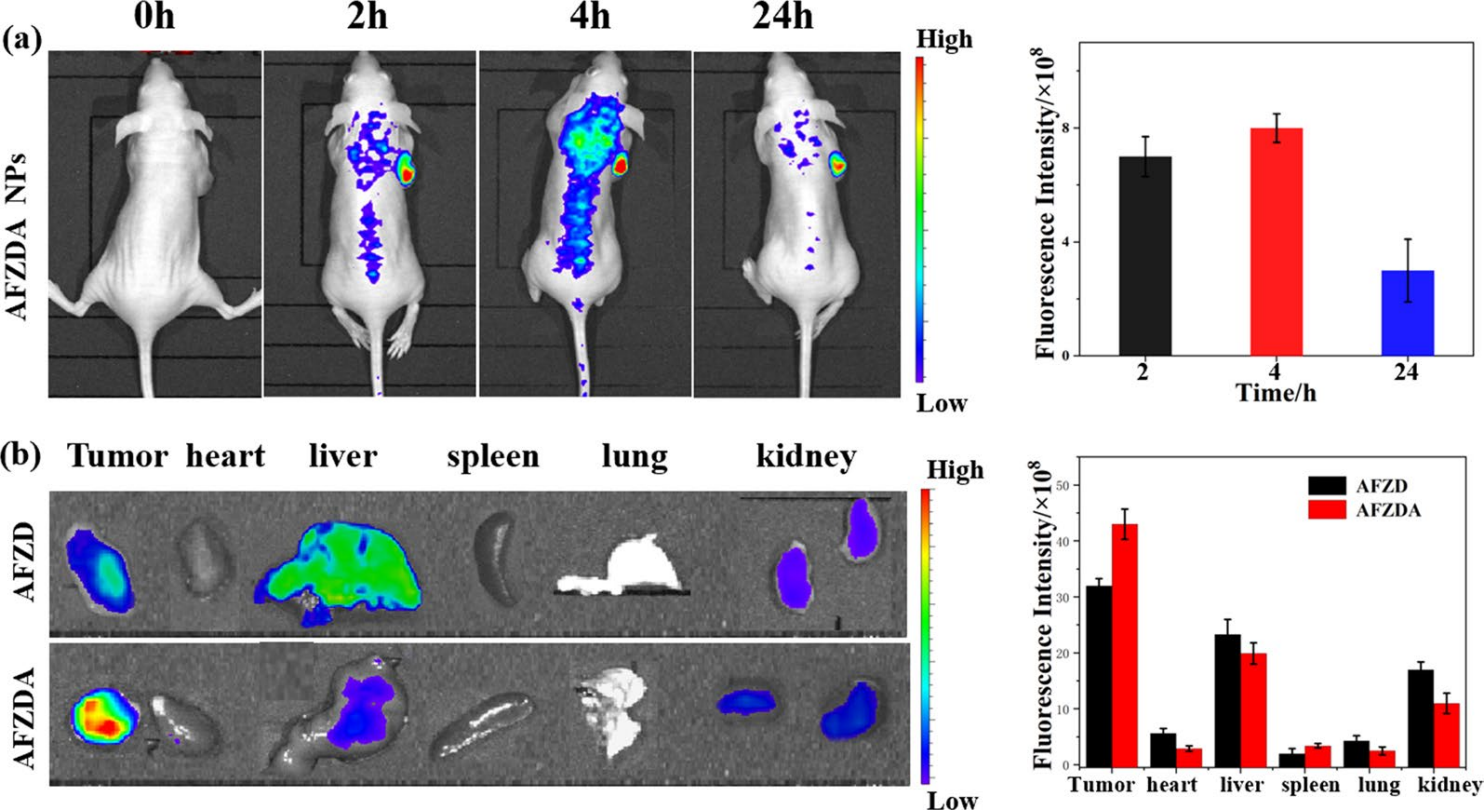
**a** In vivo fluorescence imaging of the CT26 tumor-bearing mice at 2, 4, and 24 h after intravenous injection of AFZDA NPs (left). The quantitative analysis of fluorescent signals from the tumors at 2 h, 4 h, and 24 h (n = 3, mean ± s.d.) (right). **b** Fluorescence imaging of the tumor, heart, liver, spleen, lung, kidney at 24 h after the mice were treated with AFZD NPs and AFZDA NPs, respectively. The quantitative analysis of fluorescence signals for the organs at 24 h post tail vein injection (n = 3, mean ± s.d.)

To study the distribution and metabolism of the AFZDA NPs in the mice body, the mice were administered with the nanoparticles and were fluorescently imaged in vivo. As shown in Fig. 15a, the AFZDA NPs were distributed in different parts of the mouse body at 2 h after injection, and many nanoparticles accumulated in the tumor, as reflected by the high signal intensity at the tumor site. At 4 h after injection, the distribution of the AFZDA NPs in the mouse body was more obvious, and still more nanoparticles accumulated in the tumor. At 24 h after injection, the AFZDA NPs in the mouse body dramatically decreased due to the metabolism of the nanoparticles. The fluorescence signal at the tumor site, however, was reduced only slightly, suggesting the high retention of the nanoparticles in the tumor.

Then the tumor and major organs were harvested from the mouse at 24 h after injection and were imaged fluorescently. Those harvested from a mouse treated with AFZD NPs under the same conditions were also imaged for comparison. As shown in Fig. 15b, the little signal was detected from the heart, spleen, and lung of both groups. The tumor, liver, and kidney emitted fluorescence, indicating that the nanoparticles remained in them. Compared with the AFZD NPs, more AFZDA NPs remained in the tumor, suggesting the higher retention of the AFZDA NPs that favored the tumor therapy.

**Fig. 16:**
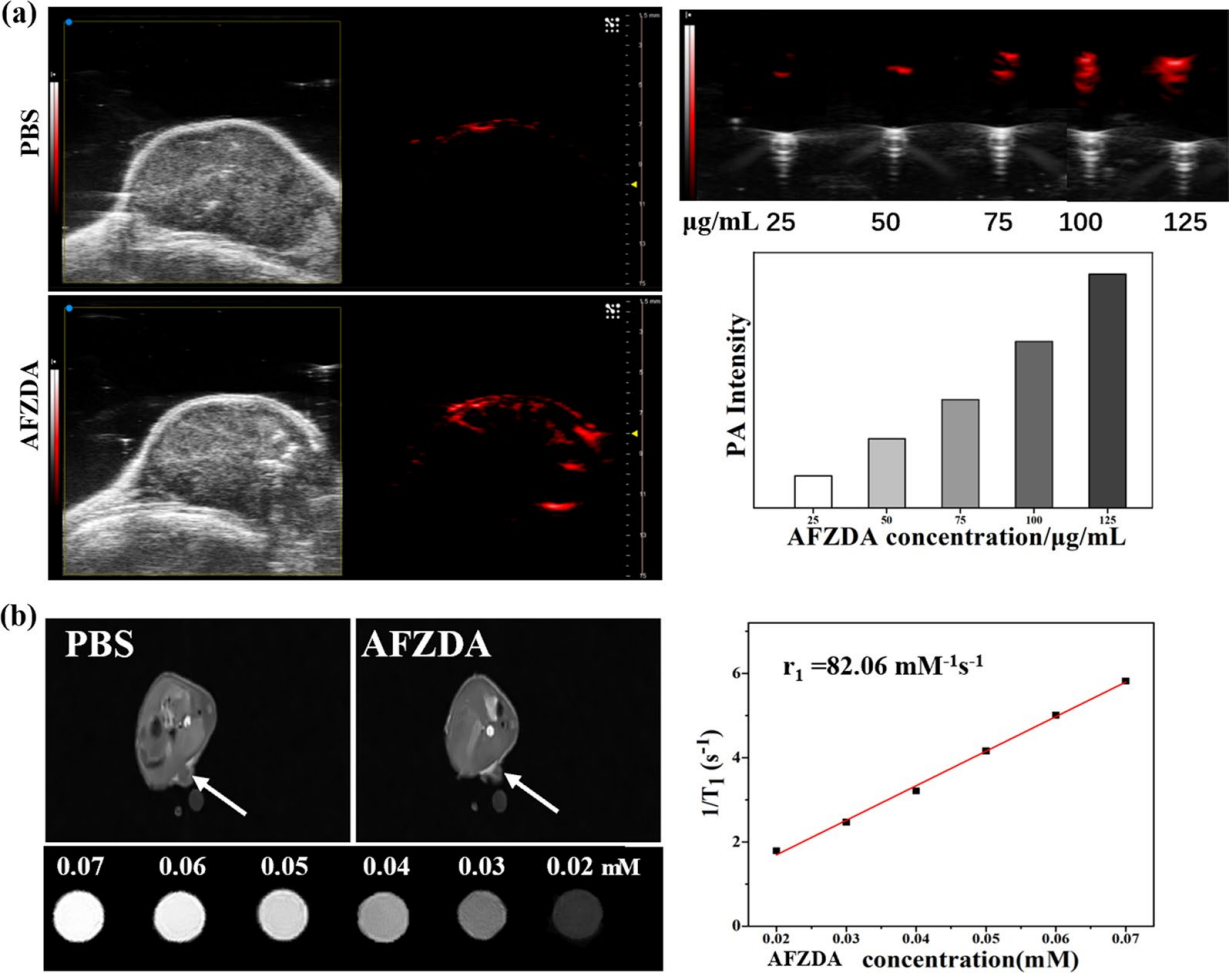
**a** The PA images of PBS and AFZDA NPs, and the quantitative PA signals of AFZDA NPs with concentrations were analyzed.  **b** The MR images of PBS and AFZDA NPs in vivo and T1-weighted magnetic resonance imaging of AFZDA NPs with concentrations in vitro, and T1 relaxation rate (r_1_) of AFZDA NPs were measured

PAI was used for monitoring the tumor microenvironment in vivo. As is shown in Fig. 16a, the signal intensity increased with increasing concentration of AFZDA NPs in vitro. Furthermore, the PA images of the AFZDA NPs-treated group showed strong structural information at the tumor site in vivo. While multimodal imaging can assist in the diagnosis and treatment of tumors, PAI enables real-time imaging to facilitate the evaluation of therapeutic effects.

The Zn_0.4_Fe_2.6_O_4_ NPs were used as an MRI contrast agent both in vitro and in vivo. At 30 min after injection of 100 μL of PBS or AFZDA NPs (0.05 mM), the tumor site of the AFZDA-treated group could be more clearly distinguished. Meanwhile, the MR signals were linearly enhanced as the AFZDA NPs concentration increased in vitro, as shown in Fig. 16b. The relaxation rate (r_1_) of AFZDA NPs was as high as 82.06 mM^−1^ s^−1^.

## Conclusion

An abnormal tumor microenvironment is a big challenge to thermotherapy and chemo-therapy due to the immunosuppression and drug penetration to the distal part of a tumor. In this work, we successfully design and fabricate AFZDA functionalized nanoparticles, which significantly enhance the thermo/chemo-therapeutic, as suggested by both in vitro cell and in vivo animal experiments. We demonstrate that AFZDA plays a critical role in the development of vascular stability and pericyte-association by balancing the expression of VEGF and ANG2. In all the cases, the vessel normalization and perfusion enhancement could be achieved in favor of the drug delivery and anti-tumor immune cell infiltration to tumor tissues. Furthermore, the immune environment is preliminarily investigated on the corrected vascular breathing with high concentrations of oxygen. The structure and function of the vascular system returned to normal, which effectively promoted the oxygen transportation. Moreover, dual-mode imaging could enhance the thermo/chemo-therapeutic efficacy. This work provides insights into the development of multifunctional systems for tumor therapy based on vessel normalization.

## References

[1] Wang J, Ni Q, Wang Y, Zhang Y, He H, Gao D, Ma X, Liang X-J (2021). Nanoscale drug delivery systems for controllable drug behaviors by multi-stage barrier penetration. J Control Release.

[2] Ji T, Kohane DS (2019). Nanoscale systems for local drug delivery. Nano Today.

[3] Pang L, Zhao R, Chen J, Ding J, Chen X, Chai W, Cui X, Li X, Wang D, Pan H (2022). Osteogenic and anti-tumor Cu and Mn-doped borosilicate nanoparticles for syncretic bone repair and chemodynamic therapy in bone tumor treatment. Bioact Mater.

[4] Holash J, Wiegand SJ, Yancopoulos GD (1999). New model of tumor angiogenesis: dynamic balance between vessel regression and growth mediated by angiopoietins and VEGF. Oncogene.

[5] Fuller AM, Olsson LT, Midkiff BR, Kirk EL, McNaughton KK, Calhoun BC, Troester MA (2019). Vascular density of histologically benign breast tissue from women with breast cancer: associations with tissue composition and tumor characteristics. Hum Pathol.

[6] Kishimoto K, Yoshida S, Ibaragi S, Yoshioka N, Okui T, Hu G-F, Sasaki A (2012). Hypoxia-induced up-regulation of angiogenin, besides VEGF, is related to progression of oral cancer. Oral Oncol.

[7] Wang JC, Li GY, Wang B, Han SX, Sun X, Jiang YN, Shen YW, Zhou C, Feng J, Lu SY, Liu JL, Wang MD, Liu PJ (2019). Metformin inhibits metastatic breast cancer progression and improves chemosensitivity by inducing vessel normalization via PDGF-B downregulation. J Exp Clin Cancer Res.

[8] Heldin C-H, Rubin K, Pietras K, Östman A (2004). High interstitial fluid pressure—an obstacle in cancer therapy. Nat Rev Cancer.

[9] Waldeland JO, Gaustad J-V, Rofstad EK, Evje S (2021). In silico investigations of intratumoral heterogeneous interstitial fluid pressure. J Theor Biol.

[10] Glicksman R, Chaudary N, Pintilie M, Leung E, Clarke B, Sy K, Hill RP, Han K, Fyles A, Milosevic M (2017). The predictive value of nadir neutrophil count during treatment of cervical cancer: interactions with tumor hypoxia and interstitial fluid pressure (IFP). Clin Transl Radiat Oncol.

[11] Chen Y, Liu X, Yuan H, Yang Z, von Roemeling CA, Qie Y, Zhao H, Wang Y, Jiang W, Kim BYS (2019). Therapeutic remodeling of the tumor microenvironment enhances nanoparticle delivery. Adv Sci.

[12] Pan W, Liu C, Li Y, Yang Y, Li W, Feng C, Li L (2021). Ultrathin tellurium nanosheets for simultaneous cancer thermo-chemotherapy. Bioact Mater.

[13] Qu B, Guo L, Ma J, Lv Y (2010). Antiangiogenesis therapy might have the unintended effect of promoting tumor metastasis by increasing an alternative circulatory system. Med Hypotheses.

[14] Marom EM, Martinez CH, Truong MT, Lei X, Sabloff BS, Munden RF, Gladish GW, Herbst RS, Morice RC, Stewart DJ, Jimenez CA, Blumenschein GR, Onn A (2008). Tumor cavitation during therapy with antiangiogenesis agents in patients with lung cancer. J Thorac Oncol.

[15] Zolochevska O, Figueiredo ML (2010). Novel tumor growth inhibition mechanism by cell cycle regulator cdk2ap1 involves antiangiogenesis modulation. Microvasc Res.

[16] Zarate MA, Rodriguez MD, Chang EI, Russell JT, Arndt TJ, Richards EM, Ocasio BA, Aranda E, Gordon EM, Yu K, Neu J, Keller-Wood M, Triplett EW, Wood CE (2017). Post-hypoxia invasion of the fetal brain by multidrug resistant staphylococcus. Sci Rep.

[17] Deng Y, Jiang Z, Jin Y, Qiao J, Yang S, Xiong H, Yao J (2021). Reinforcing vascular normalization therapy with a bi-directional nano-system to achieve therapeutic-friendly tumor microenvironment. J Control Release.

[18] Hashimoto T, Shibasaki F (2015). Hypoxia-inducible factor as an angiogenic master switch. Front Pediatr.

[19] Saeed M, Chen F, Ye J, Shi Y, Lammers T, De Geest BG, Xu ZP, Yu H (2021). From design to clinic: engineered nanobiomaterials for immune normalization therapy of cancer. Adv Mater.

[20] Yuan C-S, Deng Z-W, Qin D, Mu Y-Z, Chen X-G, Liu Y (2021). Hypoxia-modulatory nanomaterials to relieve tumor hypoxic microenvironment and enhance immunotherapy: where do we stand?. Acta Biomater.

[21] Dai X, Ruan J, Guo Y, Sun Z, Liu J, Bao X, Zhang H, Li Q, Ye C, Wang X, Zhao C-X, Zhou F, Sheng J, Chen D, Zhao P (2021). Enhanced radiotherapy efficacy and induced anti-tumor immunity in HCC by improving hypoxia microenvironment using oxygen microcapsules. Chem Eng J.

[22] Dulloo I, Phang BH, Othman R, Tan SY, Vijayaraghavan A, Goh LK, Martin-Lopez M, Marques MM, Li CW, Wang De Y, Marín Maria C, Xian W, McKeon F, Sabapathy K (2015). Hypoxia-inducible TAp73 supports tumorigenesis by regulating the angiogenic transcriptome. Nat Cell Biol.

[23] Gao M, Liang C, Song X, Chen Q, Jin Q, Wang C, Liu Z (2017). Erythrocyte-membrane-enveloped perfluorocarbon as nanoscale artificial red blood cells to relieve tumor hypoxia and enhance cancer radiotherapy. Adv Mater.

[24] de Silly RV, Derouazi M, Dietrich PY, Walker PR (2015). 45P—Hypoxia promotes IL-10 secretion by reactivated CTLs while limiting their expansion. Ann Oncol.

[25] Wang T, Zhang H, Qiu W, Han Y, Liu H, Li Z (2022). Biomimetic nanoparticles directly remodel immunosuppressive microenvironment for boosting glioblastoma immunotherapy. Bioact Mater.

[26] Chen Q, Xu L, Chen J, Yang Z, Liang C, Yang Y, Liu Z (2017). Tumor vasculature normalization by orally fed erlotinib to modulate the tumor microenvironment for enhanced cancer nanomedicine and immunotherapy. Biomaterials.

[27] Huang N, Liu Y, Fang Y, Zheng S, Wu J, Wang M, Zhong W, Shi M, Xing M, Liao W (2020). Gold Nanoparticles induce tumor vessel normalization and impair metastasis by inhibiting endothelial Smad2/3 signaling. ACS Nano.

[28] Rigamonti N, Kadioglu E, Keklikoglou I, Wyser Rmili C, Leow Ching C, De Palma M (2014). Role of angiopoietin-2 in adaptive tumor resistance to VEGF signaling blockade. Cell Rep.

[29] Zhang L, Su H, Cai J, Cheng D, Ma Y, Zhang J, Zhou C, Liu S, Shi H, Zhang Y, Zhang C (2016). A multifunctional platform for tumor angiogenesis-targeted chemo-thermal therapy using polydopamine-coated gold nanorods. ACS Nano.

[30] Fukumura D, Kloepper J, Amoozgar Z, Duda DG, Jain RK (2018). Enhancing cancer immunotherapy using antiangiogenics: opportunities and challenges. Nat Rev Clin Oncol.

[31] Keller SB, Averkiou MA (2021). The role of ultrasound in modulating interstitial fluid pressure in solid tumors for improved drug delivery. Bioconjug Chem.

[32] Xu H, Hu M, Liu M, An S, Guan K, Wang M, Li L, Zhang J, Li J, Huang L (2020). Nano-puerarin regulates tumor microenvironment and facilitates chemo- and immunotherapy in murine triple negative breast cancer model. Biomaterials.

[33] Liu L, Chen Q, Ruan C, Chen X, He X, Zhang Y, Zhang Y, Lu Y, Guo Q, Zhou W, Li C, Sun T, Jiang C (2019). Nano-engineered lymphocytes for alleviating suppressive tumor immune microenvironment, Applied. Mater Today.

[34] Zhou H-C, Chen N, Zhao H, Yin T, Zhang J, Zheng W, Song L, Liu C, Zheng R (2019). Optical-resolution photoacoustic microscopy for monitoring vascular normalization during anti-angiogenic therapy. Photoacoustics.

[35] Yeh BM, FitzGerald PF, Edic PM, Lambert JW, Colborn RE, Marino ME, Evans PM, Roberts JC, Wang ZJ, Wong MJ, Bonitatibus PJ (2017). Opportunities for new CT contrast agents to maximize the diagnostic potential of emerging spectral CT technologies. Adv Drug Deliv Rev.

[36] Wu F, Sun B, Chu X, Zhang Q, She Z, Song S, Zhou N, Zhang J, Yi X, Wu D, Wang J (2019). Hyaluronic acid-modified porous carbon-coated fe3o4 nanoparticles for magnetic resonance imaging-guided photothermal/chemotherapy of tumors. Langmuir.

[37] Xuan Y, Zhang R-Y, Zhao D-H, Zhang X-S, An J, Cheng K, Hou X-L, Song X-L, Zhao Y-D, Yang X-Q (2019). Ultrafast synthesis of gold nanosphere cluster coated by graphene quantum dot for active targeting PA/CT imaging and near-infrared laser/pH-triggered chemo-photothermal synergistic tumor therapy. Chem Eng J.

[38] El Hafny-Rahbi B, Brodaczewska K, Collet G, Majewska A, Klimkiewicz K, Delalande A, Grillon C, Kieda C (2021). Tumour angiogenesis normalized by myo-inositol trispyrophosphate alleviates hypoxia in the microenvironment and promotes antitumor immune response. J Cell Mol Med.

[39] Zhang L, Xia K, Lu Z, Li G, Chen J, Deng Y, Li S, Zhou F, He N (2014). Efficient and facile synthesis of gold nanorods with finely tunable plasmonic peaks from visible to near-IR range. Chem Mater.

[40] Ding X, Liow CH, Zhang M, Huang R, Li C, Shen H, Liu M, Zou Y, Gao N, Zhang Z, Li Y, Wang Q, Li S, Jiang J (2014). Surface plasmon resonance enhanced light absorption and photothermal therapy in the second near-infrared window. J Am Chem Soc.

[41] Leng C, Zhang X, Xu F, Yuan Y, Pei H, Sun Z, Li L, Bao Z (2018). Engineering gold nanorod-copper sulfide heterostructures with enhanced photothermal conversion efficiency and photostability. Small.

[42] Bertrand N, Wu J, Xu X, Kamaly N, Farokhzad OC (2014). Cancer nanotechnology: the impact of passive and active targeting in the era of modern cancer biology. Adv Drug Deliv Rev.

